# Hypertriglyceridemia is a dose-dependent risk factor for type 2 diabetes mellitus: a systematic review and meta-analysis

**DOI:** 10.3389/fendo.2025.1710007

**Published:** 2025-11-05

**Authors:** Luca Havelda, Eszter Ágnes Szalai, Mahmoud Obeidat, Dalma Dobszai, Dániel Sándor Veres, Tamás Kói, Emese Sipter, Szilárd Váncsa, Péter Jenő Hegyi, Maria Bucur, Anita Molnár, Klára Lara Vámossy, Péter Hegyi, Andrea Szentesi

**Affiliations:** ^1^ Centre for Translational Medicine, Semmelweis University, Budapest, Hungary; ^2^ Institute of Pancreatic Diseases, Semmelweis University, Budapest, Hungary; ^3^ Department of Restorative Dentistry and Endodontics, Semmelweis University, Budapest, Hungary; ^4^ Institute for Translational Medicine, Medical School, University of Pécs, Pécs, Hungary; ^5^ Department of Biophysics and Radiation Biology, Semmelweis University, Budapest, Hungary; ^6^ Department of Internal Medicine and Haematology, Semmelweis University, Budapest, Hungary; ^7^ Translational Pancreatology Research Group, Interdisciplinary Centre of Excellence for Research Development and Innovation, University of Szeged, Szeged, Hungary

**Keywords:** hypertriglyceridemia, HTG, type 2 diabetes mellitus, T2DM, risk factor

## Abstract

**Introduction:**

The prevalence of type 2 diabetes mellitus (T2DM) has more than doubled in the past 20 years. Most T2DM cases are preventable if risk factors are eliminated early. Hypertriglyceridemia (HTG) is also a potential but modifiable risk factor, and has a high prevalence as well. We aimed to investigate the dose-dependent effect of HTG on the development of T2DM.

**Methods:**

We carried out a systematic search in three databases (MEDLINE, Embase, and CENTRAL) on 9 November 2023. We investigated an adult population with different triglyceride levels (exposure). The outcome of interest was the development of T2DM. Pooled hazard ratio (HR), odds ratio (OR), and mean differences (MDs) with 95% confidence intervals (CIs) were calculated using a random-effects model. Risk of bias was assessed with the Quality In Prognosis Studies (QUIPS) tool. The protocol was registered in PROSPERO (CRD42023471288).

**Results:**

We identified 31,098 articles and included 101 in our meta-analysis. We found that people with HTG had more than a 1.5-fold higher risk (HR: 1.73 [1.31; 2.29]) of developing T2DM. Those who had their TG levels between 1.7 and 2.3 mmol/L had a 42% higher risk (HR: 1.42 [1.13; 1.79]), while those with TG levels above 2.3 mmol/L had an even higher risk for T2DM (HR: 1.82 [1.18; 2.87]) compared with patients with TG levels below 1.7 mmol/L. When investigating the hypertriglyceridemic waist phenotype, we found that only those with increased waist circumference had a higher risk in both sex groups among the different phenotype groups (female: HR: 2.86 [1.59; 5.14], male: HR: 3.31 [1.57; 7.27]).

**Conclusion:**

HTG is a dose-dependent risk factor for T2DM. Elevated waist circumference may have an even more important role in the development of T2DM than HTG.

**Systematic review registration:**

https://www.crd.york.ac.uk/prospero/, identifier CRD42023471288.

## Highlights

- Hypertriglyceridemia is a dose-dependent risk factor for T2DM.
- Elevated waist circumference is a risk factor for T2DM and may have an even more important role in the development T2DM than hypertriglyceridemia.
- Higher triglyceride-glucose index and triglyceride-to-high-density lipoprotein cholesterol ratio are associated with a higher risk of T2DM.

## Introduction

According to the International Diabetes Foundation, in 2021, diabetes mellitus (DM) impacted over 530 million adults worldwide, and is projected to reach 783 million by 2045 (1). Type 2 diabetes mellitus (T2DM) accounts for more than 90% of all DM cases (1) and poses substantial health and economic challenges, including rising healthcare costs, an increased burden on healthcare systems, and a higher risk of complications, such as cardiovascular disease (CVD), neuropathy, retinopathy, and nephropathy, reducing quality of life and increasing mortality (1–3). The condition is caused by genetic, environmental, and lifestyle factors, particularly poor diet, physical inactivity, and obesity (1, 4). Notably, approximately 80% of T2DM cases could be prevented by addressing modifiable risk factors, including high body mass index (BMI) and inadequate fiber intake (4).

These same modifiable risk factors can also contribute to the development of metabolic syndrome, a constellation of metabolic abnormalities including central obesity, hypertension, dyslipidemia, and impaired glucose regulation (5). This syndrome is a well-established predictor of cardiovascular morbidity and mortality, and among its components, hypertriglyceridemia (HTG) plays a key role in promoting atherogenic dyslipidemia and systemic inflammation (5–8). Furthermore, severe HTG has been linked to acute pancreatitis, underscoring the clinical importance of managing elevated triglyceride levels (9).

In addition to its established associations with CVD and pancreatitis, HTG has been identified as a potential modifiable risk factor for T2DM (10–12). Elevated triglyceride levels have been associated with increased insulin resistance (IR) and beta-cell dysfunction, both of which are pivotal in the pathogenesis of T2DM (13).

In addition to triglyceride levels, several triglyceride-related indices are increasingly used in clinical practice and research as predictors of CVD and IR (14–16).

The triglyceride-glucose index (TyG), a surrogate marker of IR, has emerged as a potential predictor of T2DM. Although it is associated with the development of T2DM, its relationship appears to be non-linear, suggesting possible thresholds influencing its predictive value (14, 17, 18).

The triglyceride-to-high-density lipoprotein cholesterol ratio (TG/HDL-C), another triglyceride-related index, has demonstrated a linear association with the incidence of T2DM, making it a straightforward risk indicator of risk escalation (19, 20, 115).

In addition, the hypertriglyceridemic waist (HTGW) phenotype, characterized by elevated triglyceride levels and abdominal obesity, has been linked to IR and is investigated as a potential indicator of T2DM. This phenotype underscores the interplay between dyslipidemia and central obesity in metabolic dysfunction (16, 21, 22).

Triglyceride itself, a simple and accessible biomarker, offers significant utility in daily clinical practice without requiring complex calculations. Similarly, triglyceride-related indices (e.g., TyG and TG/HDL-C ratio) are easily computed and available for routine screening.

However, despite extensive research into the relationship between HTG and T2DM, the dose-dependent role of triglycerides in T2DM development has not been comprehensively evaluated. The existing literature predominantly focuses on dyslipidemia as a consequence of IR or diabetes, leaving a gap in understanding the causal and dose–response relationships in the prediabetic state (23, 24).

By identifying thresholds and dose-dependent relations, clinicians could better estimate the risk of T2DM and implement targeted interventions, such as scheduling screening visits with optimal frequency and tailoring preventive measures. We hypothesize that HTG may be a dose-dependent risk factor for T2DM, and its associated indices may be predictors of T2DM. Therefore, we aimed to investigate the dose-dependent effect of HTG on the development of T2DM.

## Methods

Our systematic review and meta-analysis followed the recommendations of the Preferred Reporting Items for Systematic Reviews and Meta-Analyses (PRISMA) 2020 guidelines (25) (**Supplementary Table S1**) and the Cochrane Handbook (26). The study protocol was registered with PROSPERO (27) under accession number CRD42023471288. The protocol deviation related to the PROSPERO registration is also reported in detail in the **Supplementary Materials** (**Methods S1**). The investigation was conducted under the Systems Education education-research model run by the Centre for Translational Medicine at Semmelweis University, Budapest (28).

### Eligibility criteria and definitions

Our research aimed to investigate the effect of different triglyceride levels on the development of T2DM. We used the PEO framework to define eligibility criteria (29).

Population (P): adult patients without DM at baseline. Exposure (E): different levels of TG and triglyceride-related indices, such as TyG index, TG/HDL-C ratio, and HTGW phenotype. We used the definition of HTG, as reported in the guidelines and the articles included: >1.7 mmol/L (>150 mg/dL) (3, 4). TyG was calculated as the natural logarithm of [fasting glucose (mg/dL) × fasting triglycerides (mg/dL)/2]. Different cutoff values were used in the cohorts to categorize HTGW phenotypes; therefore, we used the category established based on the research for our analysis. In the case of HTGW, the population is divided into four groups: (1) normal TG level and normal waist circumference (control); (2) high TG level and normal waist circumference (HTNW); (3) normal TG and elevated waist circumference (NTGW); and (4) high TG level and elevated waist circumference.

Outcome (O): development of T2DM or prediabetes. Most studies used the following criteria to diagnose T2DM: fasting plasma glucose ≥7.0 mmol/L (126 mg/dL) and/or HbA1c ≥6.5% and/or the use of insulin or oral hypoglycemic agents. In some cases, the definition of newly diagnosed T2DM was different or missing; this uncertainty was taken into account during the risk of bias assessment.

Studies conducted in all publication years and all languages were included. Conference abstracts, reviews, case reports, and case series were excluded during the selection.

### Information sources

Our systematic search was conducted on 9 November 2023 in three primary databases: MEDLINE (via Pubmed), Embase, and Cochrane (CENTRAL) without filters or restrictions. We used three domains: one for T2DM, one for TG, and a third for the required study types. For each database, we used a specific search key, which can be found in **Supplementary Table S2**.

### Selection process

To remove duplicate records, we used the duplication removal tools of Endnote, followed by manual duplicate removal. We dealt with duplicate-free articles using the Rayyan tool. During the selection process, each article was reviewed by two independent authors (LH and DD/MB/AM/LV). Selection disagreements were resolved by consensus after discussion. First, title and abstract selection was performed, and then the studies included were screened by full text, where the reason for exclusion was recorded. Cohen’s kappa was calculated for each step to demonstrate the inter-rater agreement. Finally, we used the citation chaser tool for backward citation chase (Reference Chase) and dealt with the newly found articles similarly.

### Data collection process

Data were extracted independently from the eligible articles by two authors (LH and DD/AM/LV) and entered into standardized Excel spreadsheets. In case of disagreement, the conflict was resolved by discussion. If an article lacked relevant data, a letter of inquiry was sent to the corresponding author for further information.

The following data were extracted: information on the publication [e.g., year and digital object identifier (DOI) number], country, study period, number of centers, study design, and follow-up time; information on the population (e.g., sex distribution, age, and BMI), the factor measured (e.g., TyG and HTGW), the outcome investigated (e.g., prediabetes and T2DM), and their definitions and assessment methods; the value of the factor as exposure and comparator factor; the statistical results reported [e.g., multivariate hazard ratios (HRs) and univariate odds ratios (ORs)]; and methods.

### Study risk of bias assessment

Two co-authors (LH and DD/MB/AM/LV) independently assessed the risk of bias using the Quality In Prognosis Studies (QUIPS) tool (30). Disagreements were resolved by discussion, conducted separately for each outcome and factor. Further details on the Risk of Bias assessment methodology can be found in the **Supplementary Materials** (**Methods S4**).

### Data synthesis

As we assumed considerable heterogeneity among the underlying population of the reported study results by the nature of the question under investigation, random-effects models were used in a frequentist framework.

Crude and adjusted OR were used as effect size measures for categorical outcomes. To calculate the crude ORs, we extracted the total number of patients and those with the event of interest in each group separately from the studies (called “raw data”). For adjusted OR and when the studies reported the crude OR directly, we used the published value with its confidence interval (CI) in the analysis. The difference between the means (MD) was used as an effect size measure for continuous outcomes. To calculate the study MDs and pooled MD, we extracted or estimated the sample size, the mean, and the corresponding standard deviation (SD) from each study (separately for each group). For time-to-event data, we used adjusted HR with 95% CI as the main measure of effect between the two groups of interest. We summarized the findings in forest plots.

Between-study heterogeneity was also described by the between-study variance (*τ* ^2^) and Higgins and Thompson’s *I* (2–31) statistics. We directly reported the prediction interval only if the study number was over eight to ensure a meaningful estimation.

Small study publication bias was assessed by visual inspection of funnel plots and calculating Egger (for continuous, time-to-event data) or Harbord (for dichotomous) test *p*-value (32). However, we kept in mind that the diagnostic assessment of the test was limited, with less than 10 studies.

Potential outlier publications were explored using different influence measures and plots as recommended by Harrer et al. (33).

All statistical analyses were calculated by R software (34) using the *meta* (35) (v7.0.0) package for basic meta-analysis calculations and plots, and the *dmetar* (36) (v0.1.0) package for additional influental analysis calculations and plots. The package *metafor* (37) (v4.6.0) was used for the multilevel model.

For more details on calculations, data synthesis, publication bias assessment, and influential analyses, please read the detailed description in **Supplementary Methods S3**.

## Results

### Search and selection

Altogether, 31,098 articles were identified by searching three databases. After the selection process and reference checking, we included 145 studies, but only 101 articles were eligible for quantitative analysis. Because of overlapping populations, insufficient data for statistical analysis, and lack of poolable categories, the other 44 articles were included in the systematic review section and/or were used in forest plots only for visualization purposes. In the case of overlapping populations, the larger population and more recent publications were preferred, as described in more detail in the **Supplementary Materials** (**Methods S2**).

**Figure 1:**
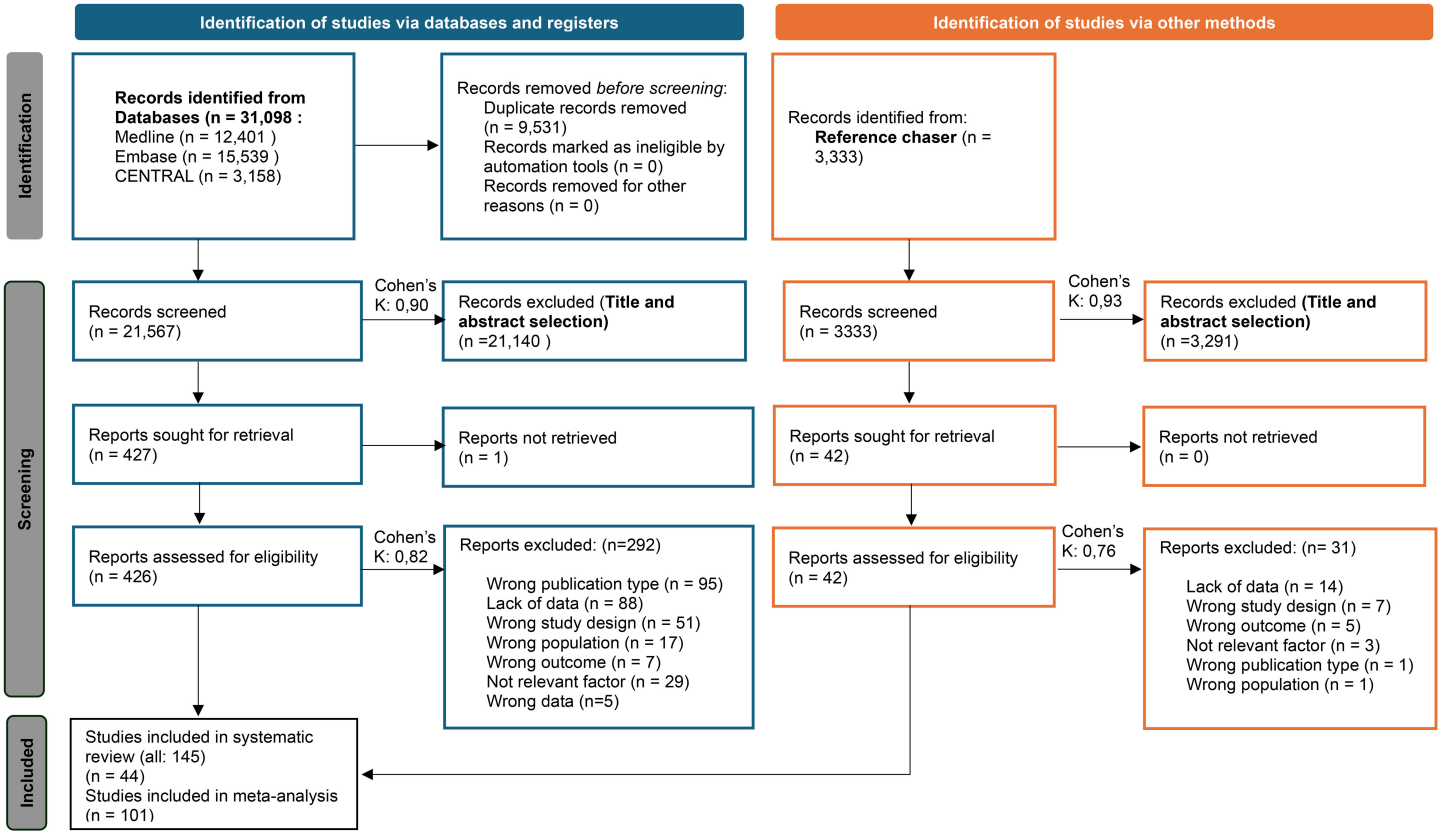
PRISMA 2020 flow diagram for new systematic reviews that included searches of databases, registers, and other sources (25).

The PRISMA flow diagram with the calculated Cohen’s kappa values (**Figure 1**) provides detailed information on the selection (25).

### Basic characteristics of studies included

The basic characteristics of the studies included are provided in the **Supplementary Materials** (**Supplementary Tables S3A, B**), separately for articles included in the quantitative synthesis and separately for articles included only in the qualitative synthesis.

### Triglyceride level

**Figure 2:**
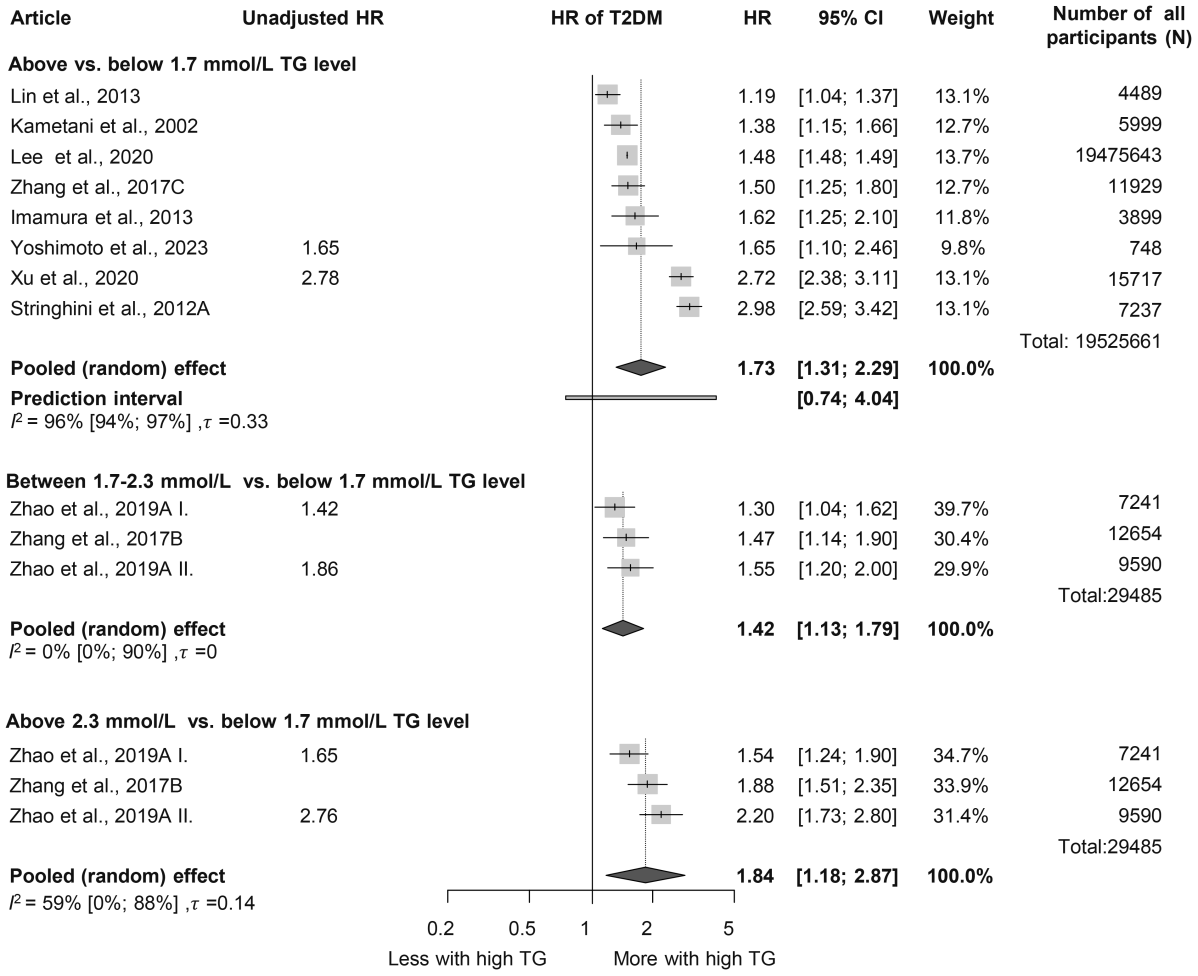
Hazard ratios for incident diabetes comparing groups with different baseline TG levels; results of three separate analyses (HR, hazard ratios; TG, triglyceride; T2DM, type 2 diabetes mellitus).

First, we analyzed patient groups with and without HTG (>1.7 mmol/L or = 150 mg/dL cutoff value). We found that according to our eight included (38–45) cohort studies with a total population of 19,525,661 participants, those with HTG at baseline had a more than 1.5-fold higher risk (HR: 1.73 [1.31; 2.29]) of the development of T2DM during the follow-up period (**Figure 2**). As we aimed to investigate the dose-dependent effect of TG level, we further analyzed two different TG level groups. According to the three included studies with a sample size of 29,485, participants with TG levels of 1.7–2.3 mmol/L had an almost 1.5-fold higher risk of incident diabetes (HR: 1.42 [1.13; 1.79]) and those with even higher TG levels (>2.3 mmol/L) had an even higher risk of developing diabetes (HR: 1.84 [1.18; 2.87]) compared with patients with TG levels below 1.7 mmol/L (**Figure 2**) (12, 46). This trend was also observed in the articles reporting OR (1.7–2.3 mmol/L: OR: 1.5 [1.01; 2.22], >2.3 mmol/L: OR: 2.44 [1.56; 3.80]) (**Supplementary Figure S2**) (47–57).

**Figure 3:**
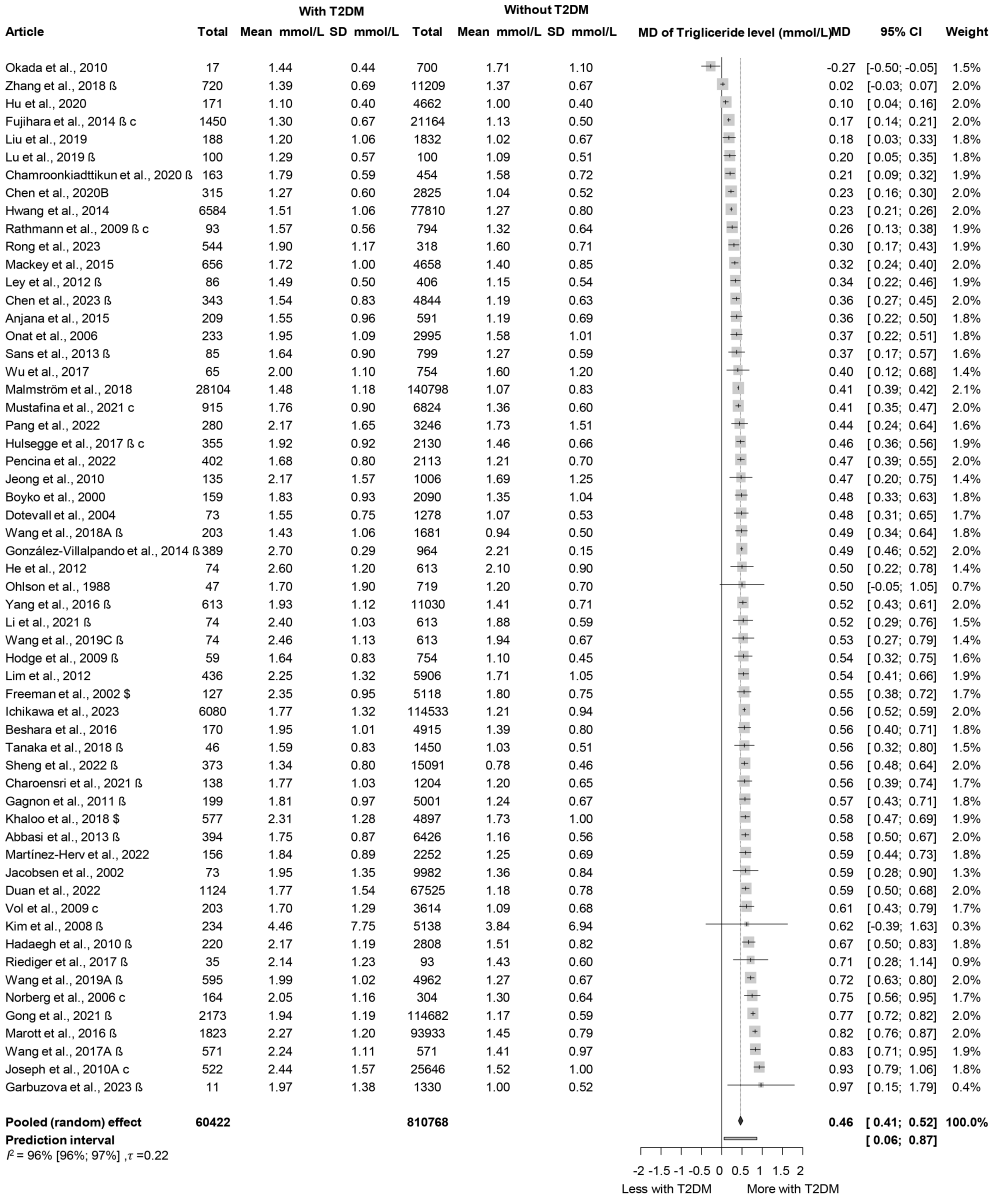
Mean differences (MD) in baseline TG levels, between patient groups with and without T2DM developed in the follow-up periods (MD, mean difference; TG, triglyceride; T2DM, type 2 diabetes mellitus; β, mean and SD are estimated mean and SD based on the quartiles in that study; c, male and female data from the same study were combined; $, mean and SD are estimated mean and SD based on the mean and SD given on the log scale variable in that study).

Analysis of baseline TG levels revealed that participants who developed diabetes had significantly higher TG levels, by nearly 0.5 mmol/L on average, compared to those who did not develop diabetes (MD: 0.46 mmol/L [0.41; 0.52 mmol/L]) (**Figure 3**, **Supplementary Figure S3**) (45, 47–50, 52, 54, 58–108). Those who developed prediabetes during follow-up had 0.31 mmol/L [0.06; 0.56 mmol/L] higher TG levels at baseline, which is a smaller difference than in those who developed T2DM (**Supplementary Figure S4**) (50, 58, 61, 89, 100, 109, 110). We also performed a subgroup analysis on the MD of TG levels in men and women; the MD was 0.50 mmol/L [0.37; 0.64 mmol/L] in women and 0.45 mmol/L [0.32; 0.58 mmol/L] in men (**Supplementary Figure S5**) (54, 63, 64, 68, 75, 80–82, 87, 90, 94, 104).

For every 50 mg/dL (0.56 mmol/L) increase in TG levels, the HR for incident diabetes was 1.08 [1.04; 1.13] based on six studies (**Supplementary Figure S6**) (39, 92, 97, 102, 111, 112) and we also visualized HRs corresponding to the SD increase in TG level in **Supplementary Figure S6** (67, 79, 81, 87, 94, 95, 113, 114). The results of articles reporting OR also suggest that a 50-mg/dL increase in TG is a risk factor for T2DM (**Supplementary Figure S7**) (47, 66, 70, 71, 84, 90, 93, 99, 106, 115–119).

### Hypertriglyceridemic waist phenotype

**Figure 4:**
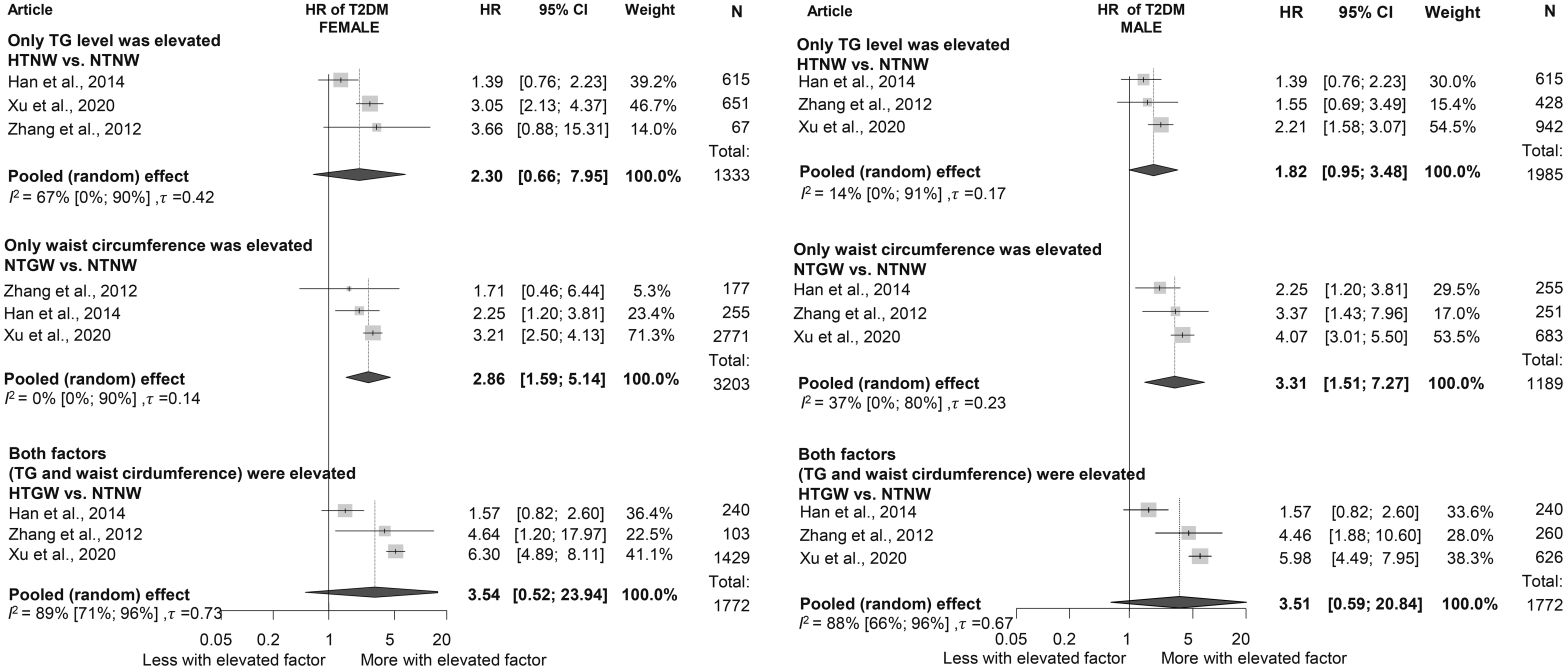
Hazard ratios for incident diabetes in groups with elevated triglyceride or/and waist circumference compared to neither elevated group [within hypertriglyceridemic waist (HTGW) phenotype groups], divided by sex (female/male); results of six separate analyses (HR, hazard ratios; TG, triglyceride; T2DM, type 2 diabetes mellitus; NTNW, normal triglyceride level and waist circumference; HTNW, elevated triglyceride level and normal waist circumference; NTGW, normal triglyceride level and elevated waist circumference; HTGW, elevated triglyceride level and waist circumference; N, number of men or women in that phenotype group).

When assessing the HTGW phenotype, we found that participants with increased waist circumference but normal TG levels had a significantly elevated HR for diabetes in both sexes (women: HR: 2.86 [1.59; 5.14]; men: HR: 3.31 [1.51; 7.27]). Among those with HTG but normal waist circumference, HRs were lower (women: HR: 2.30 [0.66; 7.95]; men: HR: 1.82 [0.95; 3.48]). Participants with both elevated TG levels and waist circumference exhibited the highest risk, with more than a 3.5-fold increased risk in both sexes; however, these results were not significant (women: HR: 3.54 [0.52; 23.94]; men: HR: 3.51 [0.59; 20.84]) (**Figure 4**) (16, 21, 43).

### Triglyceride/high-density lipoprotein ratio

**Figure 5:**
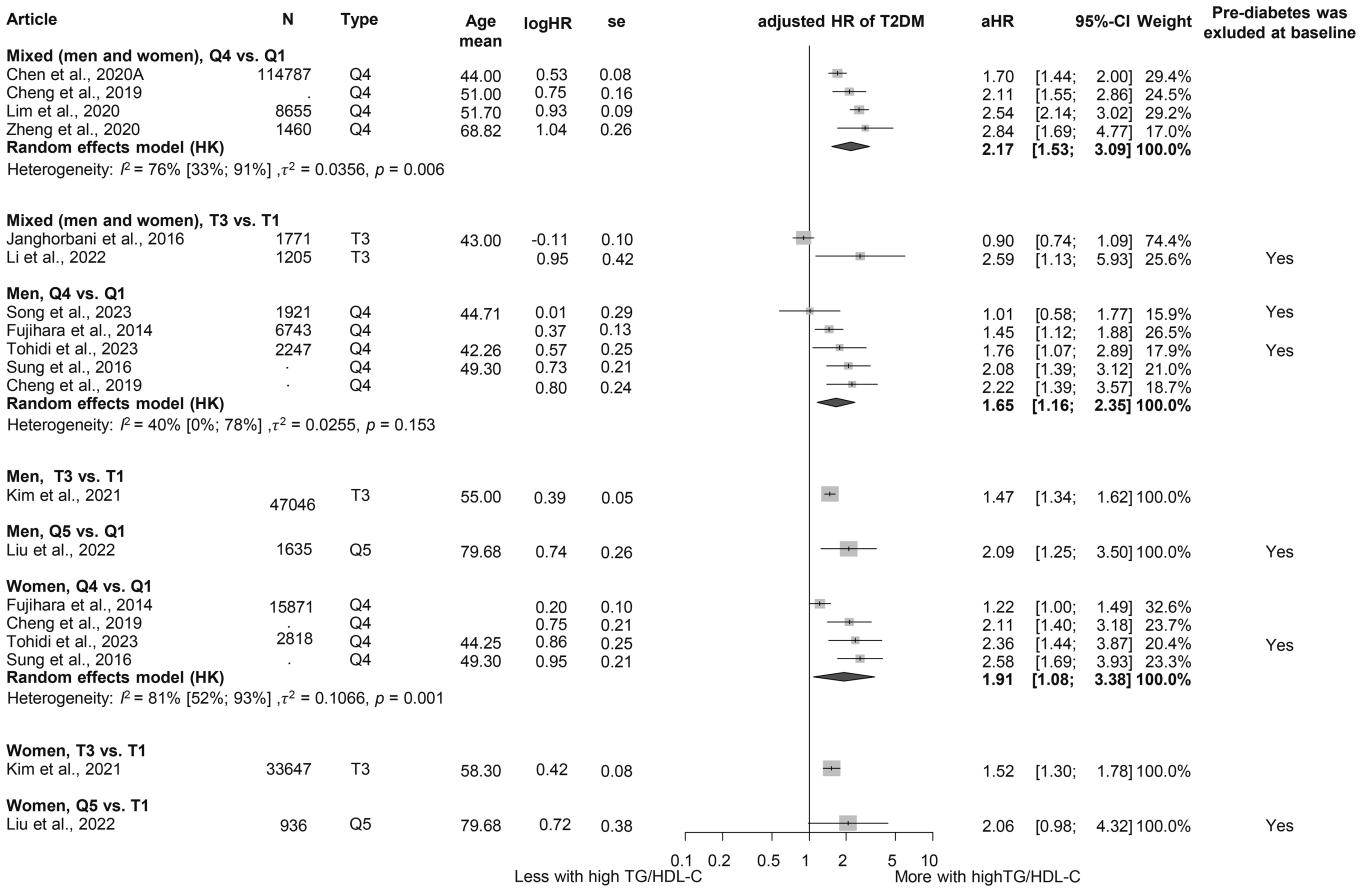
Hazard ratios for incident T2DM in groups with the highest triglyceride/high-density lipoprotein cholesterol (TG/HDL-C) ratio compared with the lowest TG/HDL-C ratio (HR, hazard ratio; T2DM, type 2 diabetes mellitus; TG/HDL-C, triglyceride/high-density lipoprotein cholesterol ratio; T1, 1st tertile; Q1, 1st quartile/1st quintile; T3, 3rd tertile; Q4, 4th quartile; Q5, 5th quintile; N, number of all participants or all male or all female participants in case of different sex groups).

We evaluated the TG/HDL-C ratio as a risk factor for diabetes. Participants in the highest TG/HDL-C quartile (Q4) had a significantly higher HR for diabetes compared to those in the lowest quartile (Q1) (HR: 2.17 [1.53; 3.09]). This finding was consistent across sex groups (women: HR: 1.91 [1.08; 3.38]; men: HR: 1.65 [1.16; 2.35]) (**Figure 5**) (3, 15, 19, 64, 96, 120–126). Baseline TG/HDL-C ratios were also significantly higher among those with diabetes (MD: 0.65 [0.20; 1.10]) (**Supplementary Figure S8**) (64, 66, 102, 120, 127). Our results show that for a one-unit increase in TG/HDL-C ratio, the risk of developing diabetes is 1.14 [1.00; 1.31] (**Supplementary Figure S9**) (15, 102, 114, 125, 128).

### Triglyceride-glucose index

**Figure 6:**
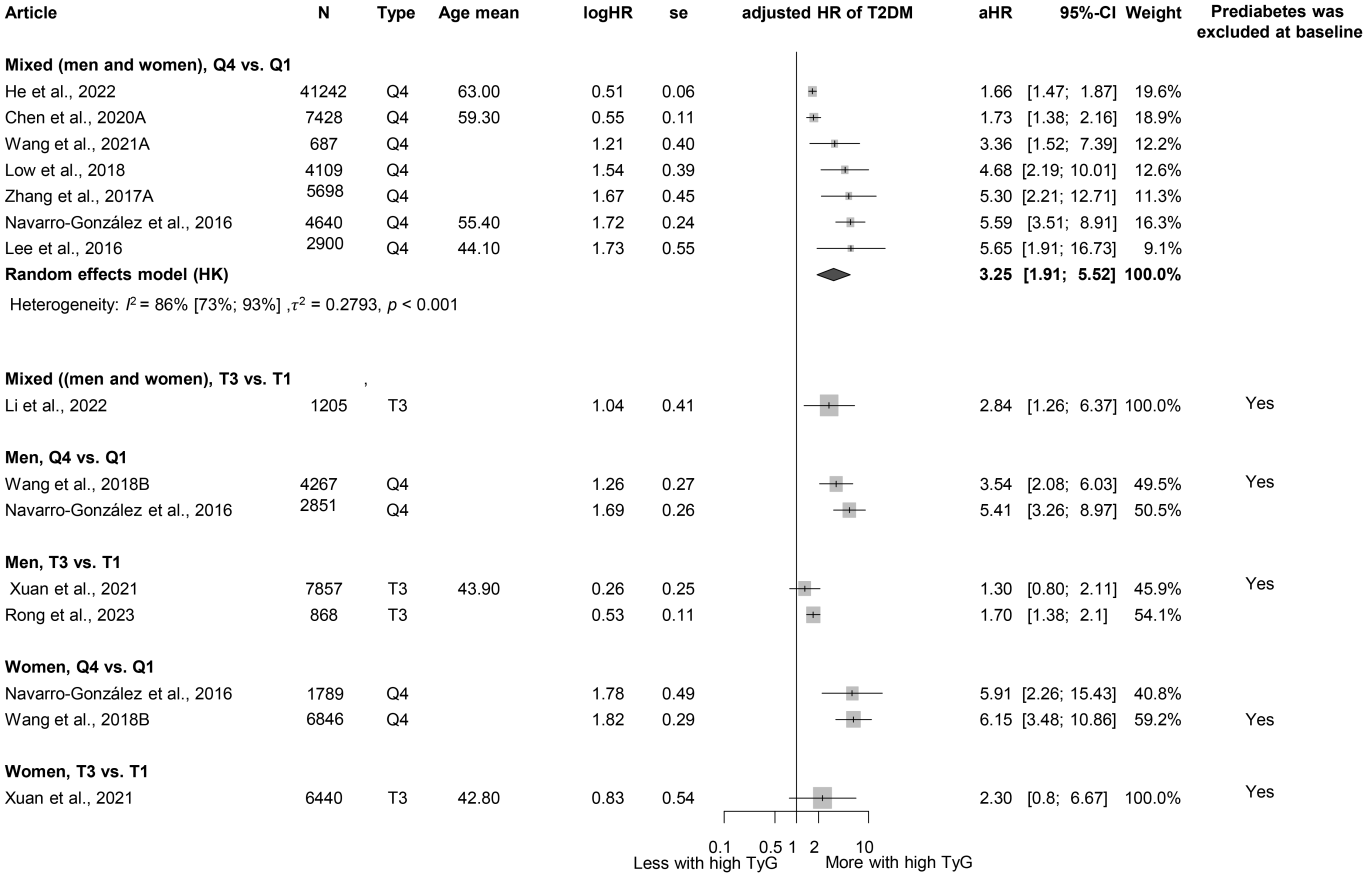
Hazard ratios for incident T2DM in groups with the highest triglyceride-glucose index (TyG) compared with groups with the lowest TyG index (HR, hazard ratio; T2DM, type 2 diabetes mellitus; TyG, triglyceride-glucose index; T1, 1st tertile; Q1, 1st quartile/1st quintile; T3, 3rd tertile; Q4, 4th quartile; N, number of all participants or all male or all female participants in case of different sex groups).

When examining TyG, we obtained similar results as for the TG/HDL-C ratio. Those in the highest TyG group by population (quartile 4) had a higher HR for diabetes than those in the lowest TyG group (Q1), with more than a threefold increased risk (HR: 3.25 [1.91; 5.52]) (**Figure 6**) (3, 17, 18, 81, 121, 127, 129–133). This risk was higher than that observed for the TG/HDL-C ratio. The MD in baseline TyG between those who did and those who did not develop T2DM was 0.30 [0.21; 0.40] (**Supplementary Figure S10**) (3, 17, 18, 60, 81, 127, 134–136). An SD increase in the Tyg index was identified as a risk factor for developing T2DM (HR: 1.36 [1.15; 1.60]), whereas for a one-unit increase in this index, the HR was 1.91 [0.61; 5.98] (**Supplementary Figure S11** (3, 18, 134, 137) and **Supplementary Figure S12** (129, 132, 133, 138)).

### Heterogeneity, risk of bias assessment, and sensitivity analysis

Heterogeneity was generally high due to the large but diverse population and different follow-up durations of the relatively large number of cohorts included. The risk of study participation, outcome definition, and statistical analysis reporting was generally low, and potential bias was observed in terms of study attrition, prognostic factor measurement, and study confounding. The detailed results for risk of bias assessment separately for articles included in the quantitative analysis and qualitative analysis are available in the **Supplementary Materials** (**Supplementary Figures S1A, B**). There was no evidence of publication bias, and the leave-one-out analyses (where it was a meaningful enough study) showed either no statistically “influential” article or the effect of the statistically “influential” article was not clinically relevant.

## Discussion

This is the first meta-analysis to examine not only individual TG levels and values but also several TG-related indices to explore the risk of diabetes in more depth. Our results suggest that triglyceride levels have a significant impact on the development of T2DM and highlight the role of different TG-related indices in this process. Our analysis suggests that the relation between TG levels and T2DM risk is dose-dependent. Although the link between HTG and T2DM has been extensively studied, most research has focused on dyslipidemia as a complication of IR or T2DM (23, 24). However, recently, with the emergence of more prediction models and research exploring T2DM risk factors, TG levels have been investigated from another perspective (139–144). In line with our own research findings, several previous and recent studies have found that higher triglyceride levels are associated with the development of DM and that HTG may act as a risk factor for T2DM (110, 145–158).

The underlying mechanisms potentially disrupt the metabolic pathways of free fatty acids (FFAs). Excess FFAs, along with resistin, tumor necrosis factor-α (TNF-α), interleukin-6, and other compounds released by enlarged adipose tissue, contribute to the development of IR, but elevated FFA levels can be triggered by increased TG production as well, further exacerbating IR (159, 160). Lipotoxicity, endoplasmic reticulum stress, and inflammation induced by dyslipidemia represent additional mechanisms that link TG abnormalities to IR (161).

Despite significant research efforts, the complex interactions between these conditions and their bidirectional influence remain to be further investigated.

While analyzing the HTGW phenotype, we found, based on the results of three cohorts, that only elevated waist circumference without HTG was a significant risk factor for T2DM in both sexes, and the highest HR was detected when both factors were elevated, consistent with previous publications (162–165). Prior studies also highlight the crucial importance of obesity and elevated adipose tissue in the development of IR and T2DM, which is partly due to the production of FFA and TNF-α and interleukins by the adipose tissue (159, 166, 167).

The TG/HDL-C ratio—an established marker of cardiovascular risk—has been shown to be associated with the incidence of IR, prediabetes, and even T2DM in previous studies (168–170). Consistent with our findings, a 2018 meta-analysis identified the TG/HDL-C ratio as an independent predictor of T2DM. The meta-analysis highlighted a dose–response relationship and reported a stronger association in female than in male patients, underscoring the importance of considering sex-specific differences in prevention strategies (15). Similarly, our analysis observed sex differences, with female patients demonstrating a higher risk of T2DM, aligning with these previous findings. In addition to the pathways already listed that lead to T2DM (such as lipotoxicity and inflammation), low HDL may directly mediate glucose, resulting in IR and T2DM (171, 172).

The TyG index has also emerged as a significant risk factor for T2DM, with an HR exceeding the HR of elevated TG/HDL-C ratio. This difference may be attributed to the inclusion of glucose—a direct marker of glucose metabolism—in the TyG index, making it a more specific indicator of glucose metabolism. The primary utility of the TyG index has previously been the risk of IR, but more recently, several studies have investigated and identified it as a risk factor for T2DM (173–176). Similar to our findings, in a previous meta-analysis, Pranata et al. also proved its impact on the development of T2DM; in their case, they found a non-linear association (14).

HTG management necessitates a multifaceted approach encompassing both lifestyle and pharmacological interventions. Lifestyle modifications, including a modest weight reduction of 5%–10%, dietary adjustments to reduce saturated fat intake, and increased dietary fiber consumption, have been shown to favorably influence triglyceride levels (177). In more severe cases, pharmacotherapy becomes essential. Statins, by inhibiting HMG-CoA reductase, limit hepatic cholesterol biosynthesis, thereby reducing triglyceride-rich lipoproteins. Fibrates activate peroxisome proliferator-activated receptors (PPARs), enhancing lipid metabolism and decreasing triglyceride levels. In addition, several novel therapeutic targets—such as apolipoprotein C3 and C2 inhibition, angiopoietin-like protein inhibition, and fibroblast growth factor analogs—emerge as promising future strategies for lowering triglycerides (8, 177).

In summary, our findings suggest that TG and TG-related indices have a strong impact on the development of T2DM. These metrics offer the advantage of being inexpensive and readily accessible; TG levels can be measured with routine blood tests, and other indices can be easily calculated using laboratory parameters. Although further research is warranted to explore their role in predicting T2DM, these markers hold promise for both healthcare systems and patients. Interventions targeting these pathways and the reduction of TG levels—such as dietary modifications, lifestyle interventions, or pharmacological agents such as fibrates—may mitigate the risk of elevated TG levels and, thus, also play a role in reducing T2DM risk.

### Strengths and limitations

The strengths of our research include the transparent methodology, the large number of patients, and the fact that we were also able to analyze different TG levels and TG-related indices as well. One of our limitations related to generalization is the high heterogeneity. Our research aimed to examine the impact of TG on the development of T2DM, with an emphasis on the exclusion of both diabetic and prediabetic populations. However, most included studies lacked this distinction, which prevents separate analyses. Owing to the limited number of studies, publication bias and influence assessment of individual studies are of limited diagnostic value.

### Implications for practice and research

Our research provides valuable insights with the potential to influence everyday clinical practices. As a result, it aligns with the principles of translational medicine, bridging the gap between scientific discovery and practical implementation (178, 179). Our results emphasize the importance of regular monitoring of accessible laboratory markers. On the basis of our findings, we recommend follow-ups to patients with elevated triglyceride levels and waist circumference to prevent diabetes. Further research should focus on effective lifestyle strategies to reduce TG levels and combat central obesity. Preventive programs are crucial to reducing the prevalence of chronic disease, and future studies should validate these markers in different populations and integrate them into risk guidelines for T2DM.

## Conclusion

HTG is a dose-dependent risk factor for T2DM. Elevated waist circumference may play an even more important role in the development of T2DM than HTG. Both high TG-HDL-C ratio and high TyG index may be predictors of T2DM.

## Data Availability

The original contributions presented in the study are included in the article/**Supplementary Material**. Further inquiries can be directed to the corresponding author.
