# Supplementary material for: Hypertriglyceridemia is a dose-dependent risk factor for type 2 diabetes mellitus: a systematic review and meta-analysis
Source: Front Endocrinol (Lausanne). 2025 Nov 5;16:1710007. doi: 10.3389/fendo.2025.1710007 (PMC12626804; doi:10.3389/fendo.2025.1710007)
Supplement: Supplementary file 1 [file SupplementaryFile1.pdf]

SUPPLEMENTARY MATERIAL

TITLE

Hypertriglyceridemia is a Dose-dependent Risk Factor of Diabetes Mellitus: A Systematic Review and Meta-analysis

AUTHORS
Luca Havelda[1,2], Eszter Ágnes Szalai[1,3], Mahmoud Obeidat[1], Dalma Dobszai[1,4], Dániel Sándor Veres[1,5], Tamás Kói[1], Emese Sipter[1,6], Szilárd Váncsa[1,2], Péter Jenő Hegyi[1,2], Maria Bucur[1], Anita Molnár[1], Klára Lara Vámossy[1,2], Péter Hegyi[1,2,4,7*] and Andrea Szentesi[1,4*]
* Contributed equally.

AFFILIATIONS

1. Centre for Translational Medicine, Semmelweis University, Budapest, Hungary
2. Institute of Pancreatic Diseases, Semmelweis University, Budapest, Hungary
3. Department of Restorative Dentistry and Endodontics, Semmelweis University, Budapest, Hungary
4. Institute for Translational Medicine, Medical School, University of Pécs, Pécs, Hungary
5. Department of Biophysics and Radiation Biology, Semmelweis University, Budapest, Hungary
6. Department of Internal Medicine and Haematology, Semmelweis University, Budapest, Hungary
7. Translational Pancreatology Research Group, Interdisciplinary Centre of Excellence for Research Development and Innovation, University of Szeged, Szeged, Hungary

CORRESPONDING AUTHOR

Andrea Szentesi, PhD

Postal address: Institute for Translational Medicine, University of Pécs, H-7624 Pécs, Szigeti út 12, Hungary

Tel.: +(36-30) 342-1481

E-mail address: szentesiai@gmail.com

Supplementary Material

TABLE OF CONTENTS

Methods S1: Detailed protocol deviation	3
Methods S2: Detailed strategy for overlapping population	3
Table S1: PRISMA Checklist	4-6
Table S2: Individualized search key in different databases	7
Methods S3: Detailed data synthesis	7-8
Table S3A: Study characteristics table (articles included in the meta-analysis)	9-13
Table S3B: Study characteristics table (articles included only in the systematic review)	13-15
Figure S1A: Risk of bias assessment table (articles included in the meta-analysis)	16
Figure S1B: Risk of bias assessment (articles included only in the systematic review)	16
Methods S4: Summary of Risk of Bias Assessment and Consensus Process	16
**Additional analysis, Forest plots**	
Figure S2: The odds ratios of incident diabetes comparing groups with different baseline TG levels	17
Figure S3: Publication Bias: The mean differences (MD) in baseline TG level, between patient groups with and without DM developed in the follow-up	18
Figure S4: Mean differences in baseline TG level, between those who developed and not developed pre-diabetes during the follow-up periods	18
Figure S5: Mean differences in baseline TG level between those who developed and those who did not develop diabetes during the follow-up periods (separately by sex groups)	19
Figure S6: The hazard ratios of incident diabetes with different TG level increase	20
Figure S7: The odds ratios of incident diabetes with different TG level increase	21
Figure S8: Mean differences in baseline TG/HDL-C ratios, between those who developed and not developed diabetes during the follow-up periods	22
Figure S9: The hazard ratios of incident diabetes in case of a one unit TG/HDL-C ratio increase	22
Figure S10: Mean differences in baseline TyG indexes, between those who developed and not developed pre-diabetes during the follow-up periods	23
Figure S11: The hazard ratios of incident diabetes in case of one SD increase in TyG index	23
Figure S12: The hazard ratios of incident diabetes in case of a one unit TyG index increase	24
Table S4: List of studies excluded at full-text screening stage, with a brief reason	24-33
References used in the Supplementary Material	33-34

Methods S1: Detailed protocol deviation

The study protocol was registered on PROSPERO (CRD42023471288). However, during our selection of articles, we came across several triglyceride-related indexes that would deepen our understanding of our original aim. Therefore, by a unified decision of the research board, we deviated from our initial PROSPERO registration. In our research framework, in our original PROSPERO registration, we would have initially only looked at different triglyceride levels for exposure. In the final meta-analysis, we included the analysis of hypertriglyceridemic-waist- -phenotype, triglyceride-glucose index, and triglyceride/high-density lipoprotein cholesterol ratio in addition to the different triglyceride levels.

Methods S2: Detailed strategy for overlapping population

In several cases of different analyses, we encountered articles that used a population from the same study or values from participants from the same cohort. In these cases, in order to avoid overlapping populations, the following criteria were used to decide which article to include in the final analysis. Priority was given to those articles with a larger population. In cases where the articles had the same population size, we decided which article to include based on the year of publication and our Risk of Bias Assessment. The excluded articles were used in the systematic review part and also in forest plots only for visualization purposes.

Supplementary Table S1. Prisma Checklist

Section and Topic	Item #	Checklist item	Item is reported
**TITLE**			
Title	1	Identify the report as a systematic review.	✓
**ABSTRACT**			
Abstract	2	See the PRISMA 2020 for Abstracts checklist.	✓
**INTRODUCTION**			
Rationale	3	Describe the rationale for the review in the context of existing knowledge.	✓
Objectives	4	Provide an explicit statement of the objective(s) or question(s) the review addresses.	✓
**METHODS**			
Eligibility criteria	5	Specify the inclusion and exclusion criteria for the review and how studies were grouped for the syntheses.	✓
Information sources	6	Specify all databases, registers, websites, organisations, reference lists and other sources searched or consulted to identify studies. Specify the date when each source was last searched or consulted.	✓
Search strategy	7	Present the full search strategies for all databases, registers and websites, including any filters and limits used.	Supplementary Table S2
Selection process	8	Specify the methods used to decide whether a study met the inclusion criteria of the review, including how many reviewers screened each record and each report retrieved, whether they worked independently, and if applicable, details of automation tools used in the process.	✓
Data collection process	9	Specify the methods used to collect data from reports, including how many reviewers collected data from each report, whether they worked independently, any processes for obtaining or confirming data from study investigators, and if applicable, details of automation tools used in the process.	✓
Data items	10a	List and define all outcomes for which data were sought. Specify whether all results that were compatible with each outcome domain in each study were sought (e.g. for all measures, time points, analyses), and if not, the methods used to decide which results to collect.	✓
	10b	List and define all other variables for which data were sought (e.g. participant and intervention characteristics, funding sources). Describe any assumptions made about any missing or unclear information.	✓
Study risk of bias assessment	11	Specify the methods used to assess risk of bias in the included studies, including details of the tool(s) used, how many reviewers assessed each study and whether they worked independently, and if applicable, details of automation tools used in the process.	✓
Effect measures	12	Specify for each outcome the effect measure(s) (e.g. risk ratio, mean difference) used in the synthesis or presentation of results.	✓, Supplementary Methods S2, S3
Synthesis methods	13a	Describe the processes used to decide which studies were eligible for each synthesis (e.g. tabulating the study intervention characteristics and comparing against the planned groups for each synthesis (item #5)).	✓, Supplementary Methods S2, S3
	13b	Describe any methods required to prepare the data for presentation or synthesis, such as handling of missing summary statistics, or data conversions.	✓, Supplementary Methods S2, S3
	13c	Describe any methods used to tabulate or visually display results of individual studies and syntheses.	✓, Supplementary Methods S2, S3
	13d	Describe any methods used to synthesize results and provide a rationale for the choice(s). If meta-analysis was performed, describe the model(s), method(s) to identify the presence and extent of statistical heterogeneity, and software package(s) used.	✓, Supplementary Methods S2, S3
	13e	Describe any methods used to explore possible causes of heterogeneity among study results (e.g. subgroup analysis, meta-regression).	✓, Supplementary Methods S2, S3
	13f	Describe any sensitivity analyses conducted to assess robustness of the synthesized results.	✓, Supplementary Methods S2, S3
Reporting bias	14	Describe any methods used to assess risk of bias due to missing results in a synthesis (arising from reporting biases).	✓

Section and Topic	Item #	Checklist item	Item is reported
assessment			
Certainty assessment	15	Describe any methods used to assess certainty (or confidence) in the body of evidence for an outcome.	NA
**RESULTS**			
Study selection	16a	Describe the results of the search and selection process, from the number of records identified in the search to the number of studies included in the review, ideally using a flow diagram.	✓
	16b	Cite studies that might appear to meet the inclusion criteria, but which were excluded, and explain why they were excluded.	✓
Study characteristics	17	Cite each included study and present its characteristics.	✓, Supplemetary Table3A and Table3B
Risk of bias in studies	18	Present assessments of risk of bias for each included study.	Supplemetary Figure S1A and S1B
Results of individual studies	19	For all outcomes, present, for each study: (a) summary statistics for each group (where appropriate) and (b) an effect estimate and its precision (e.g. confidence/credible interval), ideally using structured tables or plots.	✓, Supplemetary Fgure S2-S11
Results of syntheses	20a	For each synthesis, briefly summarise the characteristics and risk of bias among contributing studies.	✓, Supplemetary Fgure S2-S11
	20b	Present results of all statistical syntheses conducted. If meta-analysis was done, present for each the summary estimate and its precision (e.g. confidence/credible interval) and measures of statistical heterogeneity. If comparing groups, describe the direction of the effect.	✓, Supplemetary Fgure S2-S11
	20c	Present results of all investigations of possible causes of heterogeneity among study results.	✓, Supplemetary Figure S2-S11
	20d	Present results of all sensitivity analyses conducted to assess the robustness of the synthesized results.	✓, Supplemetary Figure S2-S11
Reporting biases	21	Present assessments of risk of bias due to missing results (arising from reporting biases) for each synthesis assessed.	✓, Supplemetary Figure S2-S11
Certainty of evidence	22	Present assessments of certainty (or confidence) in the body of evidence for each outcome assessed.	NA
**DISCUSSION**			
Discussion	23a	Provide a general interpretation of the results in the context of other evidence.	✓
	23b	Discuss any limitations of the evidence included in the review.	✓
	23c	Discuss any limitations of the review processes used.	✓
	23d	Discuss implications of the results for practice, policy, and future research.	✓
**OTHER INFORMATION**			
Registration and protocol	24a	Provide registration information for the review, including register name and registration number, or state that the review was not registered.	✓
	24b	Indicate where the review protocol can be accessed, or state that a protocol was not prepared.	✓
	24c	Describe and explain any amendments to information provided at registration or in the protocol.	✓. Supplementary Methods 1
Support	25	Describe sources of financial or non-financial support for the review, and the role of the funders or sponsors in the review.	✓
Competing interests	26	Declare any competing interests of review authors.	✓

Section and Topic	Item #	Checklist item	Item is reported
Availability of data, code and other materials	27	Report which of the following are publicly available and where they can be found: template data collection forms; data extracted from included studies; data used for all analyses; analytic code; any other materials used in the review.	NA

From: Page MJ, McKenzie JE, Bossuyt PM, Boutron I, Hoffmann TC, Mulrow CD, et al. The PRISMA 2020 statement: an updated guideline for reporting systematic reviews. BMJ 2021;372:n71. doi: 10.1136/bmj.n71

Supplementary Table S2. Individualized search key in different databases

Search keys used in different databases (9th of November 2023)	
**MEDLINE (via Pubmed)**	(diabetes mellitus OR "diabet*" OR "T2DM" OR "NIDDM" OR impaired glucose tolerance OR "IGT" OR "prediabetes" OR "HbA1c") **AND** ("hypertriglyceridemia" OR "hypertriglyceridaemia" OR "HTG" OR "triglycer*") **AND** ("cohort" OR "prospective" OR "longitudinal" OR "retrospective" OR "population-based" OR "population based" OR "observational" OR "registry analysis" OR "multivariate" OR "univariate")
**Embase**	**(**(diabetes mellitus OR diabet* OR T2DM OR NIDDM OR "impaired glucose tolerance" OR IGT OR prediabetes OR HbA1c) **AND** (hypertriglyceridemia OR hypertriglyceridaemia OR HTG OR triglycer*) **AND** (cohort OR prospective OR longitudinal OR retrospective OR population-based OR "population based" OR observational OR "registry analysis" OR multivariate OR univariate)**):ab,kw,ti**
**CENTRAL (Cochrane)**	(diabetes mellitus OR diabet* OR T2DM OR NIDDM OR impaired glucose tolerance OR IGT OR prediabetes OR HbA1c) **AND** (hypertriglyceridemia OR hypertriglyceridaemia OR HTG OR triglycer*) **AND** (cohort OR prospective OR longitudinal OR retrospective OR population-based OR "population based" OR observational OR "registry analysis" OR multivariate OR univariate)

Methods S3: Detailed data synthesis

We provide the following additional details on data synthesis.

If the study reported median, lower and upper quartiles instead of the mean and SD, we used the Luo and Shi methods [1,2] (as implemented in the used meta R package) to estimate the mean and SD. A few studies reported the mean and SD handling of the triglyceride concentration on a logarithmic scale. We assumed that the distribution of triglyceride level is not relevantly different from a lognormal distribution (regarding this estimation). Therefore, the Luo and Shi methods are acceptable for mean and SD estimation. We estimated the mean and SD on a "normal" scale if given on a logarithmic scale using the assumption of a lognormal distribution. If the study reported the mean and SD for independent groups separately, e.g., female and male, we used the separate data in subgroup analysis, and we also calculated the combined mean and SD and used them in the overall analysis.Pooled crude OR from raw data was calculated by the Mantel-Haenszel method [3,4].

For adjusted OR and HR logarithm transformation was performed before the meta-analysis. Inverse variance weighting method was used to calculate the pooled MD, ln (adjusted) OR and ln (adjusted) HR.

When crude OR from raw data and published crude OR with CI were both present in the analysis, we used the same approach as we used for adjusted OR.

In case of continuous covariate, the meta-analysis of the adjusted OR and HR required careful considerations. When the study categorized the values of the continuous covariate into groups based on tertiles, quartiles or quintiles, we meta-analyzed the

adjusted OR and HR values that compared the largest group with the group corresponding to the patients with the smallest covariate values. We separately pooled the tertile-, quartiles and quintiles-based results. When the continuous covariate was present without categorization in the underlying logistic or Cox regression, we separated the cases according to whether log transformation was performed and whether the coefficient corresponded to a given unit or it was given for one SD change. When the OR or HR value corresponded to a given unit, we converted it to a common unit. In the conversion, we used the fact that if a coefficient is given for a constant common unit change, then ln OR or ln HR and the corresponding SE must be divided by this constant to get the result corresponding to a common unit change.

We used a Hartung-Knapp adjustment [5,6] for CIs. This adjustment was applied only if it is more conservative then the classical one (as recommended in Jackson et al.[7] as hybrid method 2). To estimate the heterogeneity variance measure (τ^2), the Paule-Mandel method [8] for categorical, and restricted maximum-likelihood estimator for continuous outcomes (or "direct OR") was used with the Q profile method for confidence interval (recommended in Harrer et al. and Veroniki et al.[9,10]).

On the forest plots t-distribution based method used for CI of MD (and "direct OR") calculation of individual studies.

For some outcomes, the effect was reported separately for female and male in the same study and some study was conducted only on female or male. As we were interested in the effect of sex we made a subgroup analysis. We used a multilevel (3 level) meta-analysis with inverse variance weighting method to involve both the female and male data from the same article. To assess the difference between the subgroups in the multilevel models, a Wald-type test was used [9].The null hypothesis was rejected on a 5% significance level in all the performed analysis.

Potential outlier publications was explored using different influence measures - eg. we calculated dfbetas (the pooled effect size without the given study - expressed in logit scale for binary data), Cook's distances (shows how much the estimated effect size changes leaving-out the given study, but taking into consideration of how much differ the leaved-out study effect size from the pooled effect size. - typical threshold for potential influential value is 2) and hat values (the value of the hat matrix without the given study.) leaving out 1 study at a time - and plotting them following the recommandation of Harrer et. al[9] using the dmetar package. In the case of multivariate results, we calculated dfbetas, Cook's distances and hat values, leaving out 1 study at a time.

Table S3A.: Study characteristics table (articles included in the meta-analysis)

Name of article	Study type	Country	Number of patients	Male (%)	Age (Year)	BMI (kg/m2)	Follow-up time (Years)
**Abbasi et al., 2013**	prospective cohort	Netherland	6820	47.60	48.8 ± 12.5*	26 ± 4.2*	7.7 (7.4-8.0)†
**Anjana 2015**	prospective cohort	India (Chennai)	1376	41.6	N/A	N/A	9.1†
**Beshara et al., 2016**	retrospective cohort	Israel	5085	74	42.8 ± 9*	26.3 ± 3.96*	7.58 ± 1.98*
**Boyko et al., 2000**	prospective cohort	Mauritius	2605	48.9	40.4*	N/A	5¶
**Carvalho et al., 2021**	cohort	Brazil	4463	NA	35-74‡	N/A	3.7 ± 0.63*
**Chamroonkiadttikun et al., 2020**	retrospective cohort	Thailand	617	38.2	66.88 ± 10.18*	25.65 (23.79-27.95)†	9.28†
**Charoensri et al., 2021**	retrospective cohort	Thailand	1342	33.70	46 (41.0-58.8)†	23.7 (21.6-26)†	10¶
**Chen et al., 2020A**	prospective cohort	China	7428	46.5	59.3 ± 9.36*	23.31 ± 3.71*	3.4†
**Chen et al., 2023**	prospective cohort	China	6109	44.73	30-65‡	N/A	4¶
**Chen et al., 2020B**	prospective cohort	Sweden	3140	39.1	DM: 71.84 ± 5.85,<br>no-DM: 72.39 ± 5.6*	DM: 28.53 ± 4.7,<br>no-DM: 26.34 ± 4.1*	8.09 ± 2.24*
**Chen et al., 2020C**	retrospective cohort	Chinese	114787	53.2	44 ± 12.9*	23.3 ± 3.3*	3.1 ± 0.9*
**Cheng et al., 2019**	prospective cohort	Chinese	10741	38.85	N/A	N/A	6†
**Dotevall et al., 2004**	prospective cohort	Sweden	1351	0	DM: 51.6± 7.3,<br>no-DM: 49.1 ± 7.3*	DM: 28± 5,<br>no-DM: 24.3± 3.7*	18¶
**Duan et al., 2022**	prospective cohort	Netherland	68649	41.6	49.7 ± 9.5*	26.2 ± 4*	41 months ¶
**Freeman et al., 2002**	cohort	Scotland	5245	100	DM: 55.6 ± 5.7,<br>no-DM: 55.3 ± 5.5*	DM: 27.8 ± 3.7,<br>no-DM: 25.9 ± 3.1*	5¶
**Fu et al., 2021**	cohort	China	2571	63.6	79.68 ± 4.06*	23.96 ± 3.18*	3.1†
**Fujihara et al., 2014**	prospective cohort	Japan	127176	30.82	N/A	N/A	5.5*
**Gagnon et al., 2011**	prospective cohort	Australia	5200	DM: 51.3,<br>no-DM: 45.1	DM: 55.6 ± 11.9,<br>no-DM: 55.6 ± 11.9*	DM: 29.4 ± 5.6,<br>no-DM: 26.5 ± 4.5*	5¶
**Garbuzova et al., 2023**	prospective cohort	Russia	1341	46.4	37.08*	N/A	6-10¶
**Gong et al., 2021**	retrospective cohort	China	116855	53.8	44.1 ± 12.9*	23.3 ± 3.3*	3.1†

**González-Villalpando et al., 2014**	prospective cohort	Mexico	2282	NA	N/A	N/A	N/A
**Hadaegh et al., 2010**	prospective cohort	Iran	5201	41.78	>= 20‡	N/A	6.4†
**Han et al., 2014**	retrospective cohort	Korea	2900	71.65	N/A	N/A	48.7 months †
**He et al., 2012**	prospective cohort	Chinese	687	58.1	48.1 ± 6.2*	23.4 ± 2.8*	15¶
**He et al., 2022**	retrospective cohort	china	41242	47.41	63 (61-68)†	23.94 (22.21-26.23)†	N/A
**Hodge et al., 2009**	prospective cohort	Australia	813	DM: 57.6, no-DM: 47.2	DM: 59 (51.2-65.2), no-DM: 55.8 (46.5-62.5)†	DM: 30.5 (27.6-33.2), no-DM: 26.6 (24.4-29.6)†	4¶
**Hu et al., 2020**	cohort	China	4833	DM: 45, no-DM: 41.8	DM: 61 ± 7.5, no-DM: 60.8 ± 7.8*	DM: 23.2 ± 2.4, no-DM: 22.2 ± 2.4*	4.6 ± 0.4*
**Hulsegge et al., 2017**	prospective cohort	Netherland	6217	46.86	20-59‡	N/A	21¶
**Hwang et al., 2014**	retrosepctive cohort	South-Corea	84394	57.9	38.4 ± 6.8*	23 ± 2.9*	3.3*
**Ichikawa et al., 2023**	cohort	Japan	120613	76	44.2 ± 8.5*	22.9 ± 3.4*	10¶
**Imamura et al., 2013**	prospective cohort	USA	3899	42	72.7 ± 5.6*	25 ± 3.5*	11.7†
**Jacobsen et al., 2002**	case-control with prospective design	Norway	10055	DM: 61, no-DM: 51	DM: 41.4 ± 8.6, no-DM: 35.5 ± 8.6*	N/A	15¶
**Janghorbani et al., 2016**	cohort	Iran	1771	26.1	43 ± 6.5 *	N/A	7.3 ± 2.2*
**Jeong et al., 2010**	cross-sectional	Korea	1141	NA	DM: 61 ± 9, no-DM: 58 ± 11*	DM: 24.6 ± 3.2, no-DM: 23.5 ± 3.1*	5¶
**Joseph et al., 2010A**	prospective cohort	Norway	26168	47.5	25-98‡	N/A	10.8†
**Kametani et al., 2002**	prospective cohort	Japan	7222	47.77	18-85‡	N/A	4.5§
**Khaloo et al., 2018**	prospective cohort	Iran	5474	42.4	41.3 ± 13.6*	26.7 ± 4.5*	8.9†
**Kim et al., 2008**	retrospective cohort	Korea	5372	DM: 86.8, no-DM: 32.5	DM: 48.9 ± 8.1, no-DM: 46.4 ± 8.3*	DM: 25.8 ± 2.7, no-DM: 23.6 ± 2.8*	5¶
**Kuang et al., 2023**	cohort	Japan	15464	DM: 76.68, no-DM: 53.97	DM: 46 (41-53), no-DM: 42 (37-50)†	DM: 25.03 ± 3.82, no-DM: 22.04 ± 3.07*	6.13†
**Lee et al., 2020**	prospective cohort	South Korea	19475643	50.23	DM: 56.07 ± 12.81, no-DM: 44.44 ± 13.41*	DM: 24.53 ± 3.35, no-DM: 23.38 ± 3.19*	5.13†
**Lee et al., 2016**	retrospective cohort	Korea	2900	71.65	44.3 ± 6.5*	N/A	48.5 month †
**Lee et al., 2014**	prospective cohort	Korea	5354	37.7	61.6 ± 9.3*	24.2 ± 3.2*	4.6†

**Ley et al., 2012**	cohort	Canada	492	DM: 39.5, 2<br>no-DM: 42.6	DM: 31.5 ± 12.4, 2<br>no-DM: 25.4 ± 13*	DM: 29.4 ± 5.3, 2<br>no-DM: 25.4 ± 5.5*	10¶
**Li et al., 2021**	prospective cohort	China	687	58.1	48 (I44-53)†	23.2 (21.4-25.1)†	15¶
**Lim et al., 2012**	prospective cohort	Korean	6342	47.6	51.6 ± 8.7*	24 ± 3.1*	4¶
**Lim et al., 2020**	prospective cohort	Korea	8655	46.8	51.7 ± 8.8 *	N/A	12¶
**Lin et al., 2013**	cohort	USA	4489	45.2	61.1 ± 10.3*	27.5 ± 5.1*	4.75*
**Liu et al., 2011**	cohort	China	1457	DM: 95.7,<br>no-DM: 93.6	DM: 70.84 ± 6.02,<br>no-DM: 71.26 ± 6.33*	DM: 25.68 ± 3.15,<br>no-DM: 24.73 ± 3.04*	10¶
**Liu et al., 2019**	prospective cohort	China	2833	DM: 48.4,<br>no-DM: 35.5	DM: 54.85 ± 8.29,<br>no-DM: 53.42 ± 8.39*	DM: 25.42 ± 3.61,<br>no-DM: 24.69 ± 3.45*	3.1†
**Low et al., 2018**	retrospective cohort	Singapore	4109	37.11	DM: 59.3 ± 9.4,<br>no-DM: 56.4 ± 8.9*	DM: 25.3 ± 4.2,<br>no-DM: 23.5 ± 3.8*	1.4 ± 0.7*
**Lu et al., 2019**	case-control	China	200	36	DM: 59.8 ± 8,<br>no-DM: 59.8 ± 8.4*	DM: 25.2 ± 3.3,<br>no-DM: 25 ± 2.9*	4.4*
**Mackey et al., 2015**	prospective cohort	USA	5314	NA	61.6 ± 10.2*	28 ± 5.3*	7.7*
**Malmström et al., 2018**	case-control study	Sweden	296439	53	44.8 ± 13.9*	N/A	20¶
**Marott et al., 2016**	prospective cohort	Denmark	95756	DM: 54.85	DM: 65 (57-72),<br>no-DM: 57 (47-67)†	N/A	6 (0-10.9)†
**Martynez-Herv et al., 2022**	cross-sectional, prospective follow-up	Spain	2408	39.7	47.93 ± 14.76*	27.58 ± 4.74*	7.5 ± 0.6*
**Mustafina et al., 2021**	cohort	Russia	7739	43.62	57.7 ± 7.1*	28.3 ± 5.3*	13.7 ± 0.7*
**Nichols et al., 2008**	cohort	USA	46578	40.4	57.5*	29 ± 5.9*	81.0 months ¶
**Nishikawa et al., 2016**	cohort	Japan	3271	27.91	45.4*	N/A	8¶
**Norberg et al., 2006**	case-referent	Sweden	468	59.6	N/A	N/A	5.4*
**Ohlson et al., 1988**	prospective cohort	Sweden	766	100	54*	N/A	13.5¶
**Okada et al., 2010**	prospective cohort	Japan	717	DM: 47.1,<br>no-DM: 38.3	DM: 59.7 ± 13.3,<br>no-DM: 54.9 ±12.5*	DM: 25.2 ± 4.5,<br>no-DM: 25.3 ± 3.1*	10¶
**Onat et al., 2006**	prospective cohort	Turkey	3218	NA	DM: 52.8 ± 11.1,<br>no-DM: 47.7 ±12.8*	N/A	5.9*

**Orisaka et al., 2006**	cohort	Japan	2149	pre-DM: 80.9, without pre-DM: 61.18	pre-DM: 49 ± 7.2, no-pre-DM: 52 ± 9.8*	N/A	4¶
**Pang et al., 2022**	prospective cohort	China	3526	DM: 42.85, no-DM: 32.59	DM: 54.36 ± 7.64, no-DM: 52.44 ±7.36*	DM: 25.79 ± 3.03, no-DM: 24.89 ± 3.41*	5.3¶
**Park et al., 2021**	retrospective cohort	Korea	7783	47.2	51.7 ± 8.8*	24.4 ± 3*	9*
**Pencina et al., 2022**	prospective cohort	Framingham, US	2515	46.6	52.3 ± 7.9*	26.6 ± 4.6*	21.1 (11.1-23.1)†
**Quin et al., 2020**	retrospective cohort	China	116855	53.8	44.08 ± 12.93*	23.35 ± 3.3*	3.1*
**Rathmann et al., 2009**	prospective cohort	Germany	887	50.6	63.2 ± 5.4*	28.1 ± 4.0*	7¶
**Riediger et al., 2017**	cohort	Canada	128	DM: 42.9, no-DM: 45.7	DM: 37.6 ± 9.6, no-DM: 32.2 ± 8*	DM: 33.6 ± 5.4, no-DM: 30.2 ± 7*	8¶
**Rong et al., 2023**	retrospective cohort	Chinese	862	100	74 (68-79)†	25.2 ± 0.1*	20¶
**Sadeghi et al., 2014**	prospective cohort	Iran	2607	DM: 46.1, no-DM: 52.2	DM: 51.6 ± 10.6, no-DM: 47.6 ± 9.6*	DM: 27.9 ± 4.1, no-DM: 26.3 ± 4.3*	7¶
**Sans et al., 2013**	cohort	Catalonia	884	100	DM: 50.9 ± 8.1, no-DM: 49.7 ± 9*	DM: 28.1 ± 5.1, no-DM: 25.7 ± 3.1*	9.4 ± 1.4*
**Seo et al., 2011**	retrospective cohort	Korea (S)	5577	70.3	44.5 ± 5*	23.8 ± 2.8*	4¶
**Sheng et al., 2022**		Japan	15464	54.51	43.7 ± 8.9*	N/A	6.13*
**Song et al., 2023**	cohort	Japan	7684	100	N/A	N/A	6.25†
**Stringhini et al., 2012A**	prospective cohort	UK	7237	69.7	49.4 ± 6*	N/A	14.2*
**Sung et al., 2014**	cohort	Korea	1818	DM: 41.8, no-DM: 37.2	DM: 53.4 ± 11.5, no-DM: 43.3 ± 12.3*	N/A	3.7 ± 1.4*
**Sung et al., 2016**	cohort	Korea	3930	NA	49.3 ± 7.9*	24.1 ± 3*	8¶
**Tanaka et al., 2018**	prospective cohort	JAPAN	1496	37.9	55.7 ± 14.1 *	22.5 ± 2.9*	11¶
**Tohidi et al., 2023**	prospective cohort	Iran	5064	44.37	N/A	N/A	11.2†
**Wang et al., 2012**	prospective cohort	China	5408	48	46.6 ± 7.7*	N/A	15¶
**Wang et al., 2017A**	case-control	Singapore	1142	41.3	DM: 59.6 ± 6.1, no-DM: 59.7 ± 6.2*	DM: 24.8 ± 3.6, no-DM: 22.8 ± 3.3*	6-8¶
**Wang et al., 2018A**	prospective cohort	Mongolian population (China)	1884	38.11	DM: 47.71 ± 11.09, no-DM: 43.65 ± 10.90*	N/A	12¶
**Wang et al., 2019A**	case-control	Singapore	970	DM: 56.3, no-DM: 56.3	DM: 59.4 ± 5.94, no-DM: 59.4 ± 6.05*	N/A	10¶
**Wang et al., 2019C**	prospective cohort	China	687	DM: 64.84, no-DM: 57.26	DM: 49.82 ± 5.74, no-DM: 48 (IQR: 44-53)a,†	DM: 25.09 ± 3.32, no-DM: 23.19 ± 2.68*	15¶

**Wang et al., 2021A**	prospective cohort	Chinese	687	58.1	48 (44-53)†	23.4 ± 2.8*	15¶
**Wang et al., 2021B**	retrospective cohort	China	4926	53.47	44.85 ± 14.8*	N/A	10¶
**Wannamethee et al., 2010**	prospective cohort	UK	6882	DM: 57, no-DM: 50.4	DM: 68.4 ± 5.4, no-DM: 69 ± 5.5*	DM: 30.4 ± 4.8, no-DM: 26.8 ± 4.1*	7*
**Wu et al., 2017**	cohort	China	819	DM: 46, no-DM: 27	DM: 51.1 ± 10, no-DM: 47.2 ± 10.4*	DM: 28.8 ± 4.3 , no-DM: 26.2 ± 3.9*	4¶
**Xu et al., 2020**	prospective cohort	China	15717	41.82	52.7 ± 11.58*	23.32 ± 3.3*	7.77 (7.05-8.05)†
**Xuan et al., 2021**	cohort	Japan	14297	54.96	women: 42.8 ± 8.6, men: 43.9 ± 8.9*	women: 20.9 ± 2.8, men: 23.00 ± 2.9*	men: 5.88, women: 5.26 ¶
**Yang et al., 2016**	prospective cohort	China	11643	NA	50 (41-94)†	24.22 (22.01-26.66)†	6¶
**Yoshimoto et al., 2023**	cohort	Japan	748	71	54 ± 8.7*	N/A	15¶
**Yuge et al., 2023**	retrospective cohort		120613	76	44.2 ± 8.5*	22.9 ± 3.4*	6 (3-10)†
**Zhang et al., 2012**	cohort	China	2908	67.29	N/A	N/A	3¶
**Zhang et al., 2017A**	prospective cohort	China	5706	43.92	51 (40-60)†	N/A	6†
**Zhang et al., 2017B**	cohort	China	12654	39.81	N/A	N/A	6¶
**Zhang et al., 2018**	prospective cohort	China	11929	37.96	51 (41-59)†	23.98 (21.67-26.48)†	5.98†
**Zhao et al., 2019_1 cohort**	retrospective cohort	China	7241	75.80	61.49 ± 13.85*	N/A	8¶
**Zhao et al., 2019_2 cohort**	prospective cohort	China	9590	NA	N/A	N/A	8¶
**Zheng et al., 2020**	prospective cohort	China	1460	50.21	68.82 ± 8.5*	23.13 ± 3.86*	9.68 ± 6.76*

* - mean (and standard deviation), † - median, ‡- range, § - average, ¶ - not specified, DM group: Those who developed diabetes mellitus during the follow-up period. no-DM: Those who did not develop diabetes mellitus during the follow-up period.

Table S3B.: Study characteristics table (articles included only in the systematic review and used only for visualisation in forest plots)

Name of article	Study type	Country	Number of patients	Male (%)	Age (Years)	BMI (kg/m2)	Follow-up time (Years)
**Asgari et al., 2023**	prospective cohort	Iran	1835	45.6	56.02 ± 7.89*	28.27 ± 4.38*	9.3 (8.4-10.1)†
**Balkau et al., 2008**	prospective cohort	France	3817	48.8	30-64‡	N/A	9¶
**Burchfiel et al., 1995**	cohort	Hawaii	7210	100	45-68‡	N/A	6¶
**Carlsson et al., 2014**	cross-sectional	Sweden	1026	100	71 ± 0.6*	N/A	6†

**Chen et al., 2021**	retrospective cohort	Chine	6918	46.5	N/A	N/A	4¶
**Harati et al., 2009**	prospective cohort	Iran	3307	42	42 ± 13*	26.7 ± 4.7*	6†
**He et al., 2013**	prospective cohort	China	687	58	N/A	N/A	15¶
**Hwang et al., 2015**	prospective cohort	USA (japanese americans population)	406	51	51.6 ± 11.8*	24.2 ± 3.3*	10¶
**Janghorbani et al., 2015**	prospective cohort	Iran	1477	24.3	43 ± 0.03*	28.9 ± 0.11*	6.9 ± 1.7 *
**Joseph et al., 2011**	prospective cohort	Norway	16168	47.5	25-98‡	N/A	10.8†
**Kim et al., 2021**	retrospective cohort	Korea	80693	58.3	40-79 ‡	N/A	5.9†
**Lee et al., 2018**	prospective cohort	Korea	7708	47.17	men: 51.4 ± 8.6, women 52 ± 8.9*	men: 24.1 ± 2.9, women: 24.7 ± 3.2*	10¶
**Ley e al., 2008**	prospective cohort	Canadian	492	DM: 39.5, 2 no-DM: 42.6	DM: 31.5 ± 12.4, 2 no-DM: 25.4 ± 13*	DM: 29.4 ± 5.3, 2 no-DM: 25.4 ± 5.5*	10¶
**Ley et al., 2010**	cohort	USA (Canadian population)	492	DM: 39.5, 2 no-DM: 42.6	DM: 31.5 ± 12.4, 2 no-DM: 25.4 ± 13*	DM: 29.4 ± 5.3, 2 no-DM: 25.4 ± 5.5*	10¶
**Li et al., 2020**	retrospective cohort	China	201298	54.3	42.08 ± 12.67*	23.19 ± 3.34*	3.12 ± 0.94*
**Li et al., 2022**	prospective cohort, cross-sectional	China	1205	NA	24-75‡	N/A	12.17†
**Liu et al., 2021**	retrospective cohort	China	7791	33.54	56.03 ± 7.82 *	25.74 ± 3.41*	4¶
**Liu et al., 2022**	retrospective cohort	China	2571	63.6	79.68 ± 4.06*	N/A	3.2¶
**Magliano et al., 2008**	cross-sectional	Australia	5842	45.7	50.9*	26.7*	5¶
**Mann et al., 2010**	cohort	US	5329	46.7	61.6 ± 10.2*	28 ± 5.3*	4.75*
**Miljkovic et al., 2022**	cohort	Usa, Denmark	1585	42.8	60.1 ± 8*	27 ± 4.7*	7.9 ± 1.1*
**Navarro-González et al., 2016**	prospective cohort	Spain	4820	60.81	women: 55.7 ± 13.68, men: 53.57 ± 12.97*	N/A	8.84 ± 4.39*
**Olafsdottir et al., 2009**	retrospective cohort	Iceland	2251	42.47	50*	N/A	26*
**Owei et al., 2016**	prospective cohort	Tennessee-US	335	46.15	44.8 ± 10.4*	30.2 ± 7.26*	5.5± 2.62*
**Park et al., 2020**	retrospective cohort	Korea	4285	44	51.5 ± 8.9 *	22.3 ± 1.8*	9-12¶
**Peng et al., 2021**	prospective cohort	China	7329	47.14	58.77 ± 9.4*	23.4 ± 3.72*	3.4*

**Perry et al., 1995**	prospectove cohort	England,	7577	100	40-59‡	N/A	12.8*
**Ren et al., 2017**	prospective cohort	China	12086	37,21	51 (41-59) median†	N/A	6¶
**Saijo et al., 2022**	retrospective cohort	Japan	46001	83.84	42.06 ± 6.09*	23.09 ± 3.28*	10¶
**Shimodaria et al., 2023**	retrospective cohort	Japan	24604	59.34	N/A	N/A	5¶
**Skretteberg et al., 2013**	prospective cohort	Norway	1962	100	40-59‡	N/A	35¶
**Szili-Torok et al., 2022B**	prospective cohort	Netherlands	2085	42.5	47.4 (39-57.4)†	35.1 (22.9-27.2)†	11.4 (10.9-12.2)†
**Tirosh et al., 2008**	cohort	Israel	13953	100	32.4*	N/A	10.5*
**Tohidi et al., 2018**	prospective cohort	Iran	4419	42	40.6 ± 13.2*	26.6 ± 4.5*	12†
**Valdes et al., 2007**	prospective cohort	Spain	634	43.7	51.5± 12.4 *	27.2 ± 4.4*	N/A
**Valdés et al., 2008**	prospective cohort	Spain	464	NA	50.1 ± 12.5*	26.7 ± 4.3*	6.3*
**Wang et al., 2017B**	prospective cohort	Singapore	39528	46.61	N/A	N/A	4¶
**Wang et al., 2018B**	prospective cohort	China	11113	38.4	50 (41-59)†	23.91 (21.62-26.37)†	6†
**Wang et al., 2019B**	cohort	China	5557	DM: 73.95,<br>no-DM: 58.26	DM: 53.14 ± 12.7,<br>no-DM: 43.26 ± 13.11*	DM: 25.16 ± 3.04,<br>no-DM: 23.46 ± 3.15*	3¶
**Yang et al., 1995**	prospective cohort	China	432	50.92	N/A	N/A	6¶
**Yatsuya et al., 2018**	prospective cohort	Japan	3540	100	47.8 ± 7*	N/A	12.2†
**Young et al., 2019**	cohort	Canada	1254	38.52	N/A	N/A	5¶
**Zhang et al., 2016**	cohort	China	11564	37.82	51 (42-59)†	N/A	6¶
**Zhou et al., 2016**	prospective cohort	China	383	34.76	N/A	N/A	4¶

* - mean (and standard deviation), † - median, ‡- range, § - average, ¶ - not specified, DM group: Those who developed diabetes mellitus during the follow-up period. no-DM: Those who did not develop diabetes mellitus during the follow-up period.

Figure S1A.: Risk of bias assessment table (articles included in the meta-analysis)

Figure created by using Risk-of-bias VISualization (robvis)[11]

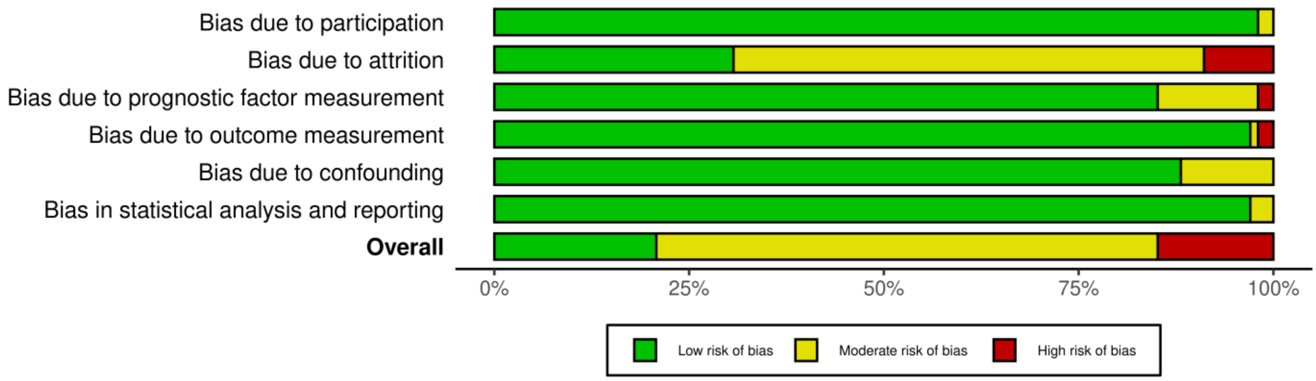

Figure S1B.: Risk of bias assessment (articles included only in the systematic review)

Figure created by using Risk-of-bias VISualization (robvis)[11]

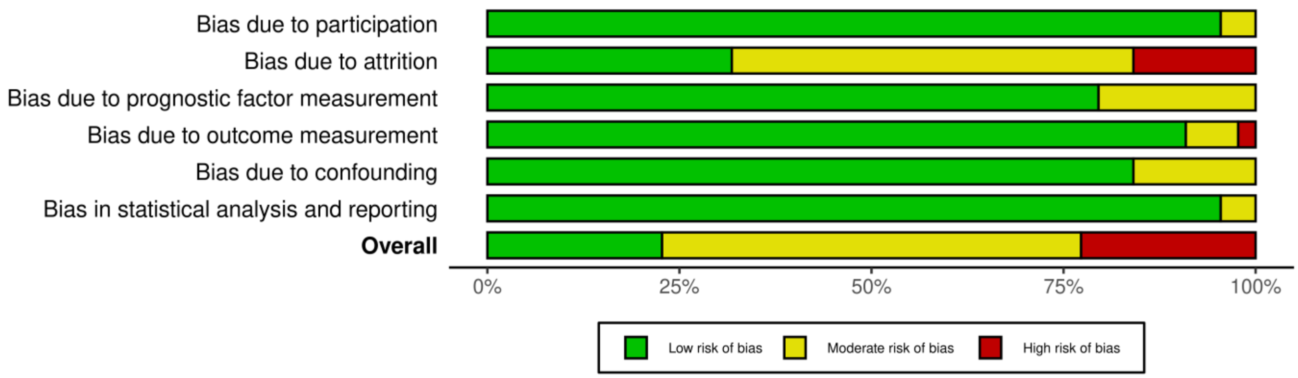

Methods S4: Summary of Risk of Bias Assessment and Consensus Process

For the Risk of Bias (RoB) Assessement the Quality in Prognostic Studies (QUIPS) tool was applied, with standardized written guidance provided by LH to all investigators (DD/MB/AM/LV) to ensure methodological consistency. Each study was independently evaluated, and the RoB ratings were subsequently compared between LH and the co-investigators. The overall disagreement rate remained low across most domains, including *study participation*, *outcome measurement*, *study confounding*, and *statistical analysis*, while greater variability was observed for *Prognostic factor measurement* and *Study attrition*. In the former, disagreements primarily arose from the reporting of the calculation of triglyceride-related indices (e.g., TG/HDL-C ratio, TyG index). In some studies, it was described in detail, whereas in others, this information was missing. Some assessors, therefore, rated such cases as “moderate risk,” while others, relying on general knowledge, assigned a “low risk” rating. The latter domain (*Study attrition*) showed the widest discrepancies due to differences in the interpretation and identification of attrition data. Some investigators sought specific figures or tables for attrition data, while others accepted textual references. In several cases, relevant information was found only in the supplementary materials, which not all co-investigators consulted. These discrepancies were clarified and resolved through discussion.

Figure S2: The odds ratios of incident diabetes comparing groups with different baseline TG levels

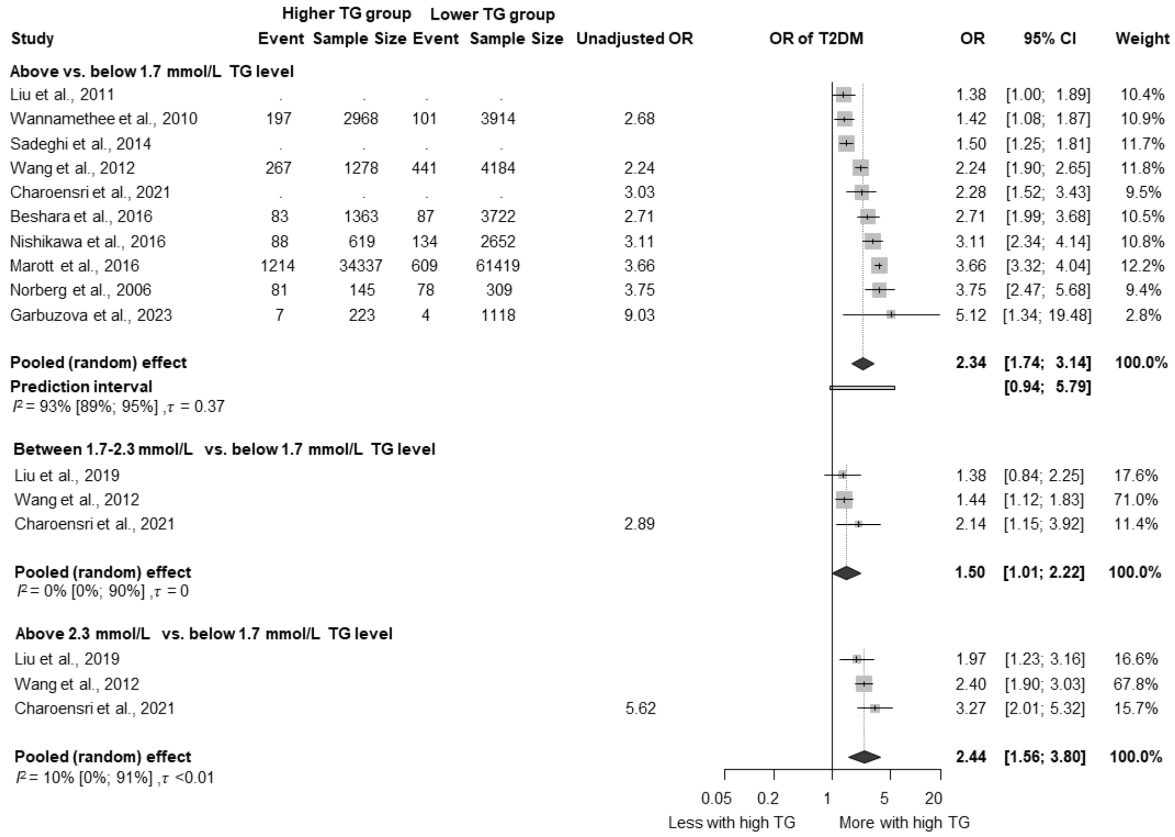

Results of three different analysis, OR – Odds ratio, TG – triglyceride, T2DM – Type 2 Diabetes Mellitus

Figure S3: Publication Bias: The mean differences (MD) in baseline TG level, between patient groups with and without DM developed in the follow-up

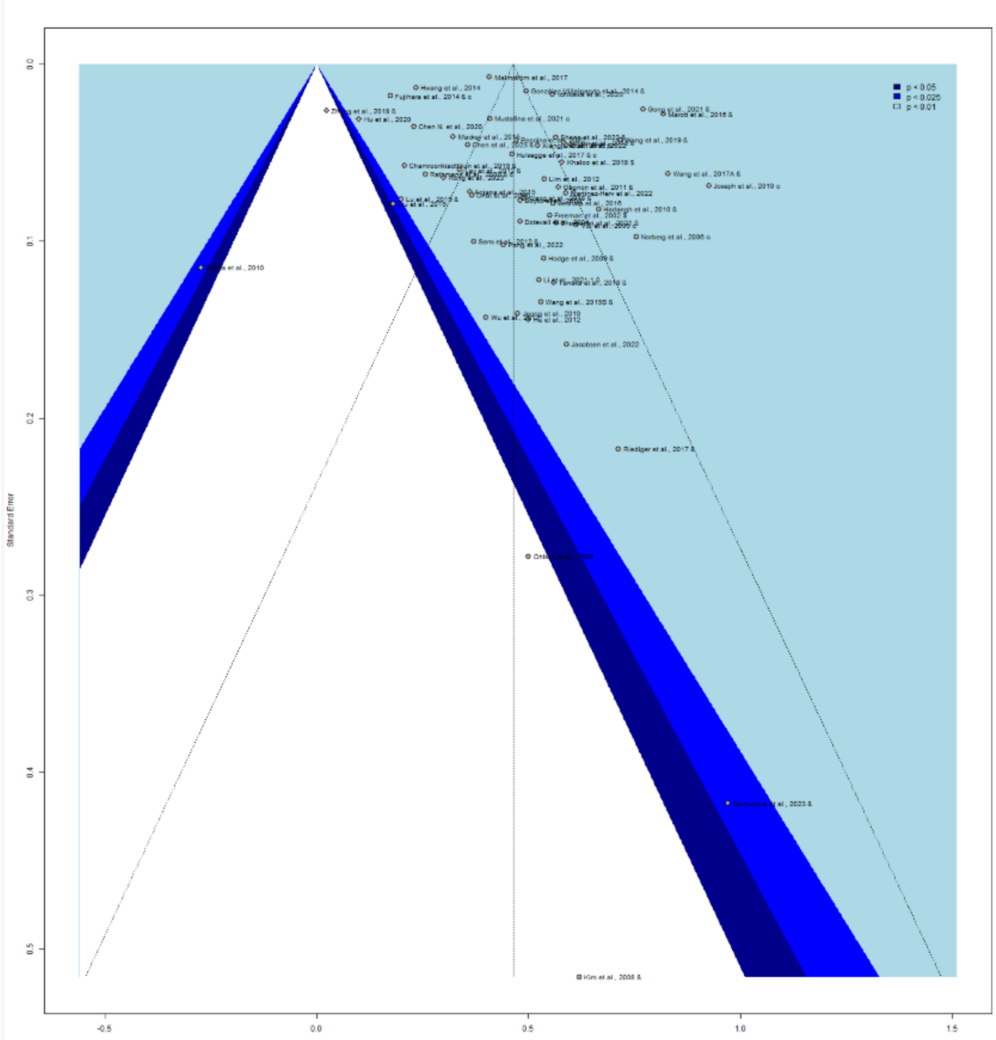

Egger's test p-value is: 0.1993.

Figure S4: Mean differences in baseline TG level, between those who developed and not developed pre-diabetes during the follow-up periods

	With pre-DM			Without pre-DM						
Study	Total	Mean mmol/L	SD mmol/L	Total	Mean mmol/L	SD mmol/L	MD of Trigliceride level (mmol/L)	MD	95% CI	Weight
Ohlson et al., 1988	75	1.20	0.40	430	1.20	0.70		0.00	[-0.11; 0.11]	15.2%
Anjana et al., 2015	277	1.28	0.60	591	1.19	0.69		0.09	[0.00; 0.18]	15.5%
Liu et al., 2019	813	1.15	0.78	1832	1.02	0.67		0.13	[0.07; 0.19]	15.8%
Chen et al., 2023 ß	376	1.55	0.68	4844	1.19	0.63		0.36	[0.29; 0.43]	15.7%
Wang et al., 2021B	442	1.88	1.58	4484	1.38	0.96		0.50	[0.35; 0.65]	14.6%
Gong et al., 2021 ß	9773	1.67	0.89	107028	1.14	0.67		0.54	[0.52; 0.55]	16.1%
Orisaka et al., 2006	21	2.00	1.26	2128	1.13	0.69		0.87	[0.33; 1.41]	6.9%
**Pooled (random) effect**	**11777**			**121337**				**0.31**	**[0.06; 0.56]**	**100.0%**
I^2 = 98% [97%; 99%] , τ = 0.24										

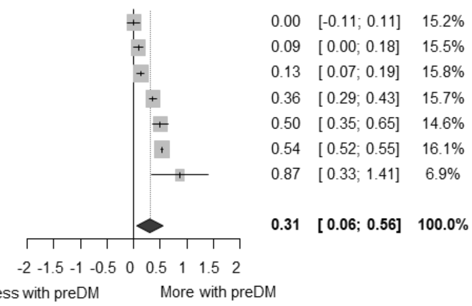

MD – mean difference, TG – triglyceride, pre-DM – prediabetes

Figure S5: Mean differences in baseline TG level between those who developed and those who did not develop diabetes during the follow-up periods (separately by sex groups)

Study	With T2DM Total	With T2DM Mean mmol/L	With T2DM SD mmol/L	Without T2DM Total	Without T2DM Mean mmol/L	Without T2DM SD mmol/L	MD	95%-CI	Weight
**male**									
Fujihara et al., 2014 ß	572	1.41	0.6689	6171	1.27	0.5932	0.14	[0.08; 0.19]	16.5%
Rathmann et al., 2009 ß	60	1.54	0.6070	389	1.40	0.7441	0.14	[-0.03; 0.31]	11.0%
Rong et al., 2023	544	1.90	1.1662	318	1.60	0.7133	0.30	[0.17; 0.43]	6.9%
Sans et al., 2012 ß	85	1.64	0.9043	799	1.27	0.5941	0.37	[0.17; 0.57]	6.1%
Mustafina et al., 2021	328	1.70	0.9000	3048	1.30	0.6000	0.40	[0.30; 0.50]	14.8%
Hulsegge et al., 2017 ß	194	1.97	1.0456	1164	1.57	0.7422	0.40	[0.25; 0.55]	12.2%
Vol et al., 2009	140	1.79	1.4500	1723	1.26	0.8000	0.53	[0.29; 0.77]	8.5%
Freeman et al., 2002 $	127	2.35	0.9540	5118	1.80	0.7514	0.55	[0.38; 0.72]	6.4%
Joseph et al., 2010A	308	2.55	1.7800	12123	1.75	1.1300	0.80	[0.60; 1.00]	10.1%
Norberg et al., 2006	97	2.30	1.3000	182	1.30	0.6000	1.00	[0.73; 1.27]	7.5%
**Pooled (random) effect**	**2455**			**31035**			**0.45**	**[0.31; 0.60]**	**100.0%**
**Prediction interval**								**[-0.01; 0.92]**	
**female**									
Fujihara et al., 2014 ß	878	1.24	0.6683	14993	1.07	0.4448	0.17	[0.12; 0.21]	17.1%
Rathmann et al., 2009 ß	33	1.63	0.4644	405	1.24	0.5208	0.39	[0.23; 0.56]	11.4%
Norberg et al., 2006	67	1.70	0.8000	122	1.30	0.7000	0.40	[0.17; 0.63]	8.5%
Mustafina et al., 2021	587	1.80	0.9000	3776	1.40	0.6000	0.40	[0.32; 0.48]	15.6%
Dotevall et al., 2004	73	1.55	0.7500	1278	1.07	0.5300	0.48	[0.31; 0.65]	6.6%
Hulsegge et al., 2017 ß	161	1.87	0.7479	966	1.34	0.5197	0.54	[0.42; 0.66]	13.2%
Vol et al., 2009	63	1.50	0.7800	1891	0.93	0.5000	0.57	[0.38; 0.76]	9.7%
Hadaegh et al., 2010 ß	220	2.17	1.1940	2808	1.51	0.8159	0.67	[0.50; 0.83]	6.7%
Joseph et al., 2010A	214	2.29	1.1800	13523	1.31	0.8100	0.98	[0.82; 1.14]	11.2%
**Pooled (random) effect**	**2296**			**39762**			**0.50**	**[0.35; 0.65]**	**100.0%**
**Prediction interval**								**[0.04; 0.97]**	

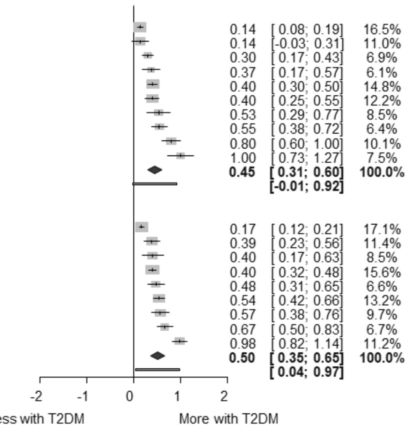

Total/2= 93% [0%; 98%]
Between study I2: 70% [0%; 59%] Within study I2:23% [0%; 39%]
Omnibus test for subgroup difference: p= 0.4521

MD – mean difference, TG – triglyceride, T2DM – Type 2 Diabetes Mellitus, ß - mean and SD are estimated mean and sd based on the quartiles in that study, $ - mean and SD are estimated mean and SD based on the mean and sd given on log scale variable in that study)

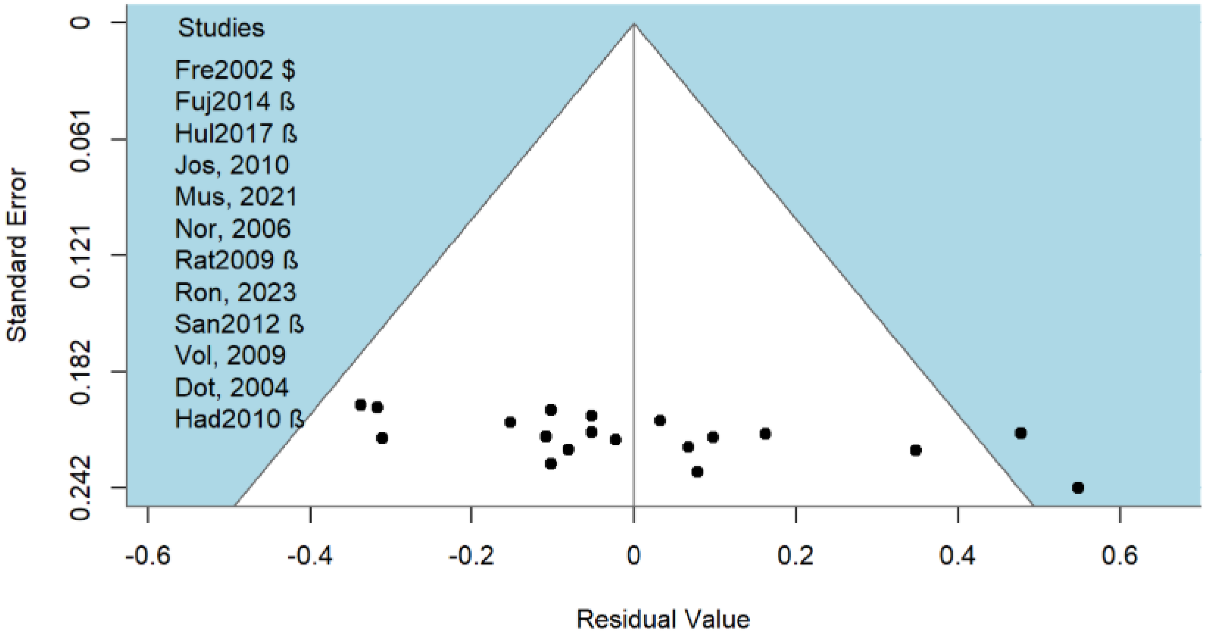

Figure S6: The hazard ratios of incident diabetes with different TG level increase

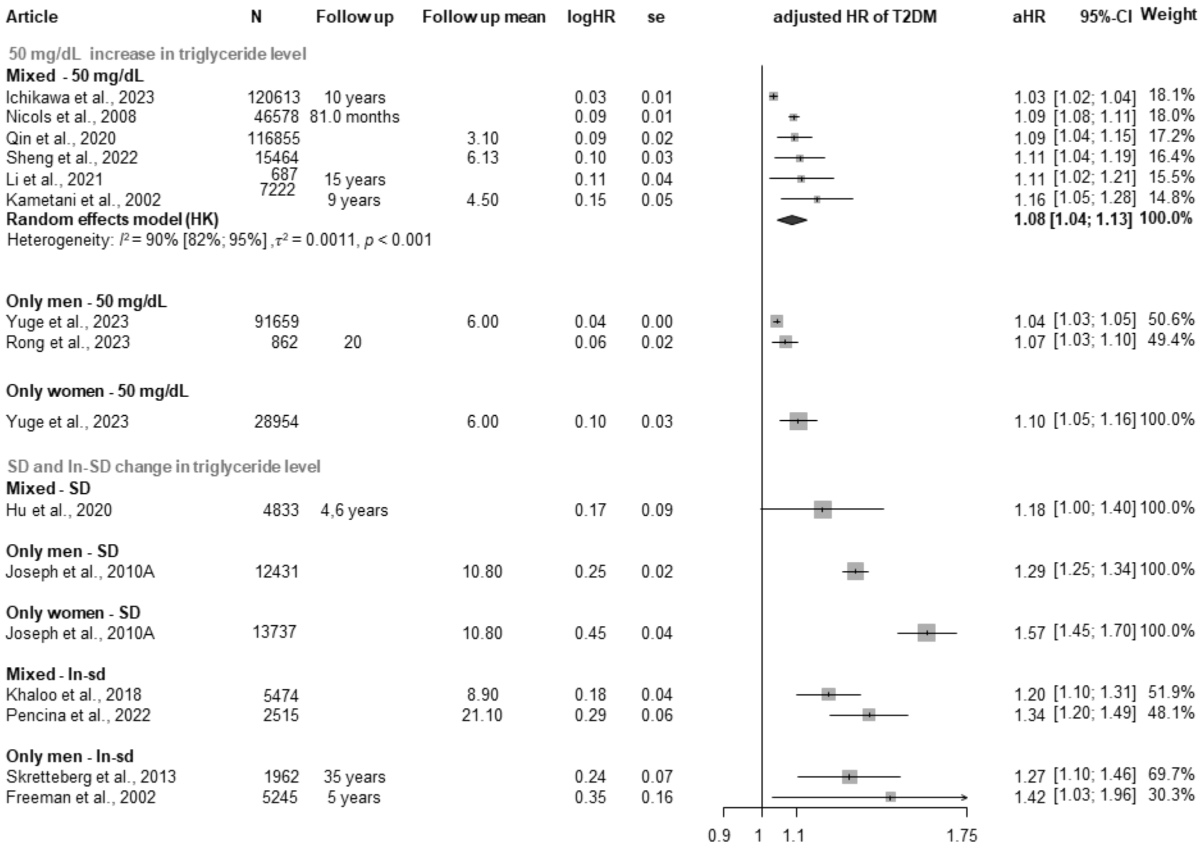

HR – hazard ratio, TG – triglyceride, T2DM – Type 2 Diabetes Mellitus, SD – standard deviation

Figure S7: The odds ratios of incident diabetes with different TG level increase

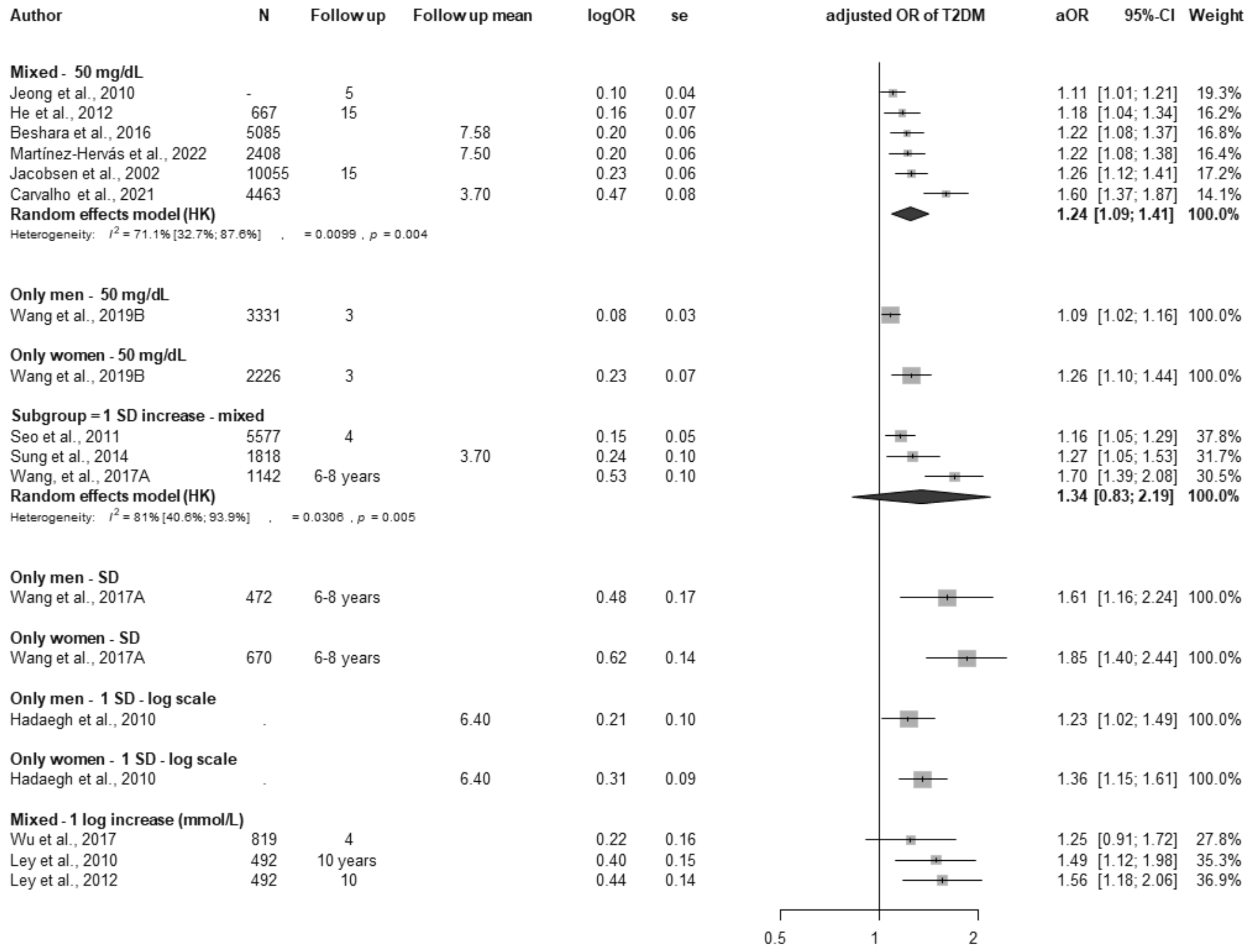

OR – odds ratio, TG – triglyceride, SD – standard deviation, T2DM – Type 2 Diabetes Mellitus

Figure S8: Mean differences in baseline TG/HDL-C ratios, between those who developed and not developed diabetes during the follow-up periods

Study	With T2DM Total	With T2DM Mean	With T2DM SD	Without T2DM Total	Without T2DM Mean	Without T2DM SD	MD	95%-CI	Weight
**Men**									
Fujihara et al., 2014	572	1.40	1.4000	6171	1.20	1.0000	0.20	[0.08; 0.32]	52.3%
Wang et al., 2018B	188	1.88	1.3600	4081	1.45	1.0000	0.43	[0.23; 0.63]	47.7%
Heterogeneity: I^2 = 74% [0%; 94%] ,τ^2 = 0.0196, p = 0.05									
**Women**									
Fujihara et al., 2014	878	1.10	0.9000	14993	0.80	0.7000	0.30	[0.24; 0.36]	51.8%
Wang et al., 2018B	251	1.86	1.1900	6593	1.38	0.9700	0.48	[0.33; 0.63]	48.2%
Heterogeneity: I^2 = 79% [10%; 95%] ,τ^2 = 0.0128, p = 0.03									
**Mixed (men and women)**									
Fujihara et al., 2014	1450	1.22	1.1332	21164	0.92	0.8196	0.30	[0.24; 0.36]	27.1%
He et al., 2012	74	2.29	1.2200	613	1.73	0.9500	0.56	[0.27; 0.85]	20.6%
Sheng et al., 2022	373	1.55	1.5400	15091	0.71	0.7000	0.84	[0.68; 1.00]	25.0%
Janghorbani et al., 2016	261	4.90	0.2000	1510	4.00	0.0800	0.90	[0.88; 0.92]	27.4%
**Random effects model**	**2158**			**38378**			**0.65**	**[0.20; 1.10]**	**100.0%**
Heterogeneity: I^2 = 99% [99%; 99%] ,τ^2 = 0.0784, p < 0.01									

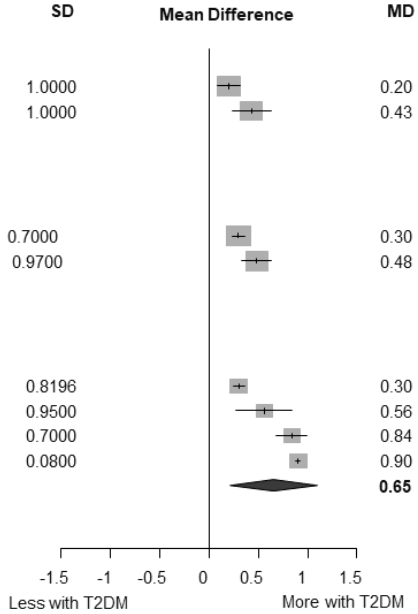

MD–mean difference, TG/HDL-C – triglyceride/high-density cholesterol ratio, T2DM – Type 2 Diabetes Mellitus, SD – standard deviation

Figure S9: The hazard ratios of incident diabetes in case of a one unit TG/HDL-C ratio increase

Author	N	Follow up	Follow up mean	logHR	se	aHR	95%-CI	Weight
**Mixed (men and women)**								
Yuge et al., 2023	120613	.	6.00	0.03	0.00	1.03	[1.02; 1.04]	28.7%
Chen et al., 2020C	114787	.	3.10	0.15	0.02	1.16	[1.10; 1.22]	26.3%
Sheng et al., 2022	15464	.	6.13	0.17	0.05	1.18	[1.08; 1.29]	21.8%
Cheng et al., 2019	.	.	6.00	0.22	0.04	1.25	[1.16; 1.35]	23.3%
**Random effects model (HK)**						**1.14**	**[1.00; 1.31]**	**100.0%**
Heterogeneity: I^2 = 94.7% [89.4%; 97.3%], τ^2 = 0.0065, p <0.001								
**Women**								
Yuge et al., 2023	28947	.	6.00	0.05	0.01	1.05	[1.02; 1.08]	35.6%
Tohidi et al., 2023	2817	.	11.20	0.08	0.02	1.08	[1.05; 1.12]	35.3%
Cheng et al., 2019	.	.	6.00	0.24	0.05	1.27	[1.16; 1.39]	29.1%
**Random effects model (HK)**						**1.12**	**[0.88; 1.43]**	**100.0%**
Heterogeneity: I^2 = 87.3% [63.9%; 95.5%]								
**Men**								
Yuge et al., 2023	91666	.	6.00	0.03	0.00	1.03	[1.02; 1.04]	64.7%
Tohidi et al., 2023	2247	.	11.20	0.05	0.01	1.05	[1.02; 1.08]	32.7%
Cheng et al., 2019	.	.	6.00	0.17	0.07	1.19	[1.04; 1.37]	2.6%
**Random effects model (HK)**						**1.04**	**[0.97; 1.12]**	**100.0%**
Heterogeneity: I^2 = 65.9% [0.0%; 90.2%]								

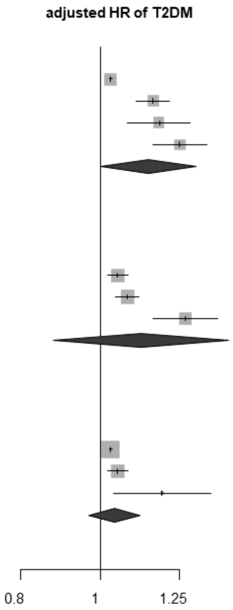

HR – hazard ratio TG/HDL-C – triglyceride/high-density cholesterol ratio, T2DM – Type 2 Diabetes Mellitus, N- number of participant in the group (total: both women and men, or just men or just women)

Figure S10: Mean differences in baseline TyG indexes, between those who developed and not developed pre-diabetes during the follow-up periods

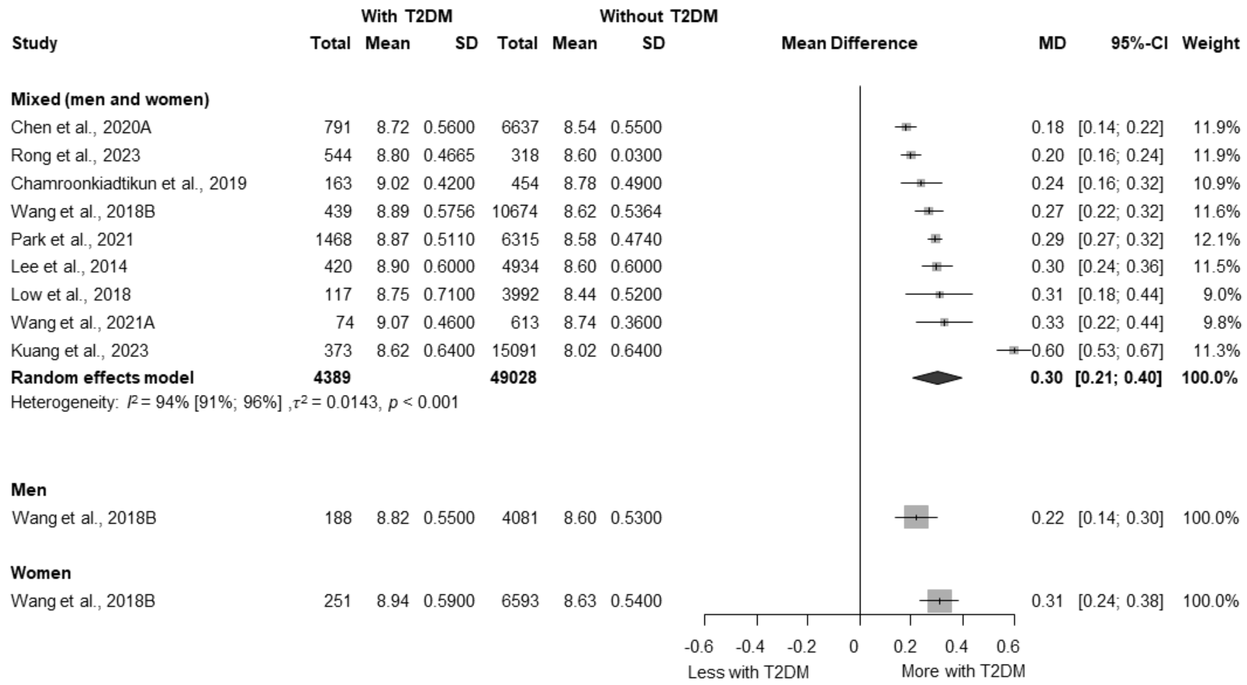

MD–mean difference, TyG – triglyceride-glucose index, T2DM – Type 2 Diabetes Mellitus, SD – standard deviation

Figure S11: The hazard ratios of incident diabetes in case of one SD increase in TyG index

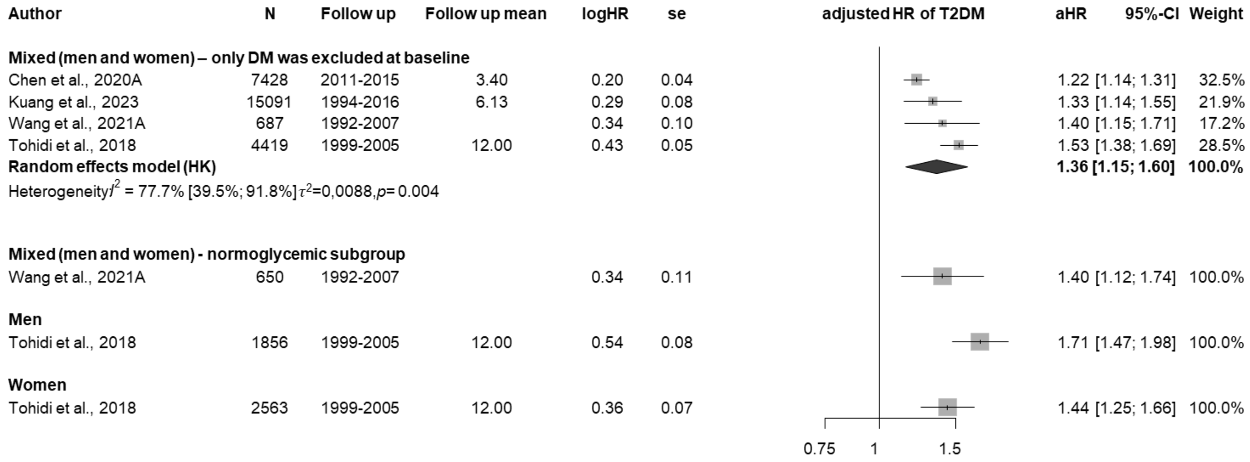

HR – hazard ratio, TyG index – triglyceride-glucose index, T2DM – Type 2 Diabetes Mellitus, N- number of participant in the group (total: both women and men, or just men or just women)

Figure S12: The hazard ratios of incident diabetes in case of a one unit TyG index increase

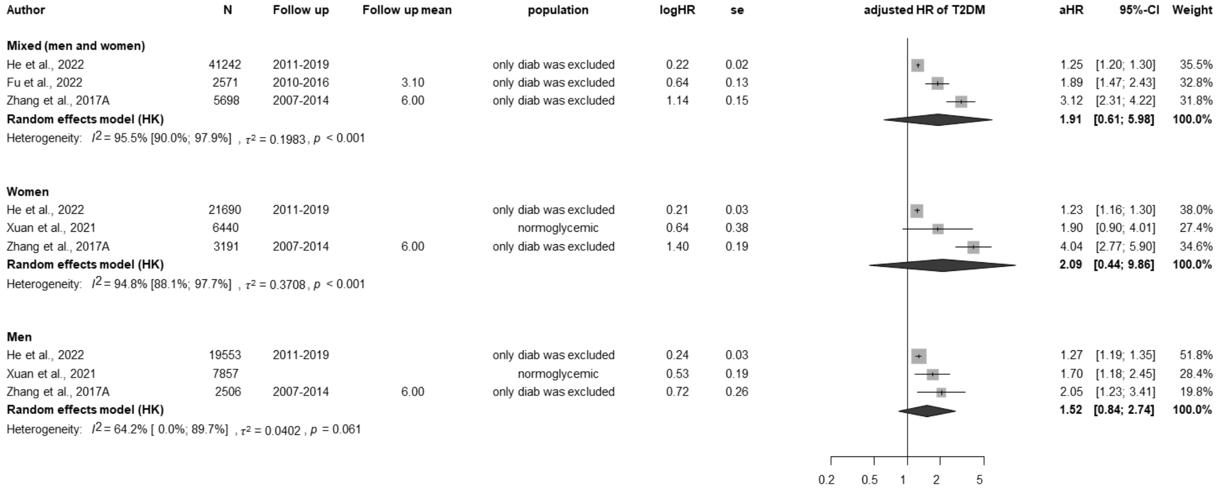

HR – hazard ratio, TyG index – triglyceride-glucose index, T2DM – Type 2 Diabetes Mellitus, N- number of participant in the group (total: both women and men, or just men or just women)

Table S4. List of studies excluded at full-text screening stage, with a brief reason

Studies exluded at full-text screening stage (Identification via Databases and Registers) n=292		
**First author and year of publication**	**Title**	**Reason**
Abdella et al., 1996	Impact of the 1997 American Diabetes Association criteria on classification of glucose intolerance among Kuwaitis below 50 years of ag	wrong study design
Abutineh et al., 2019	Circulating WBC, RBC, and hemoglobin level in relation to metabolic syndrome and incident prediabetes in a biracial cohort of normoglycemic subjects with parental type 2 diabetes	wrong publication type
Aekplakorn et al., 2006	A Risk Score for Predicting Incident Diabetes in the Thai Population	wrong data
Aguayo et al., 2016	Prevalence of diabetes mellitus and impaired glucose metabolism in the adult population of the Basque Country, Spain	wrong study design
Ahmad et al., 2018	Triglycerides, triglyceride-rich lipoprotein subfractions and genetic predisposition for type 2 diabetes in the Women Genome's Health Study (WGHS)	wrong publication type
Ahola-Olli et al., 2019	Circulating metabolites and the risk of type 2 diabetes-a prospective study of 10,938 young adults from four Finnish cohorts	wrong publication type
Akehi et al., 2010	Serum γ-glutamyltransferase, triglyceride and total cholesterol are possible prediabetic risk markers in young Japanese men	wrong study design
Al Akl et al., 2023	The triglyceride glucose-waist-to-height ratio outperforms obesity and other triglyceride-related parameters in detecting prediabetes in normal-weight Qatari adults: A cross-sectional study	wrong study design
Al-Mawali., 2021	Prevalence and risk factors of diabetes in a large community-based study in the Sultanate of Oman: STEPS survey 2017	wrong study design
Alva et al., 2017	Identifying risk for type 2 diabetes in different age cohorts: does one size fit all?	lack of data
Angelin et al., 2010	Serum fibroblast growth factor 21 and triglycerides independently predict the development of type 2 diabetes	wrong publication type
Asgari et al., 2023	External validation of the American prediction model for incident type 2 diabetes in the Iranian population	lack of data
Austin et al., 1995	Prospective Study of Small LDLs as a Risk Factor for Non-Insulin Dependent Diabetes Melltius in Elderly Men adn Women	wrong population
Bener et al., 2019	Vitamin D and Elevated Serum Uric Acid as Novel Predictors and Prognostic Markers for Type 2 Diabetes Mellitus	wrong study design
Berg et al., 2021	Pre- and post-diagnostic blood profiles of chlorinated persistent organic pollutants and metabolic markers in type 2 diabetes mellitus cases and controls; a pilot study	lack of data
Beshara et al., 2013	Hypertriglyceridemia a risk factor for diabetes: A large cohort study	wrong publication type
Bhole et al., 2010	Serum Uric Acid Levels and the Risk of Type 2 Diabetes: A Prospective Study	lack of data

Bouillon et al., 2013	Diabetes Risk Factors, Diabetes Risk Algorithms, and the Prediction of Future Frailty: The Whitehall II Prospective Cohort Study.	wrong outcome
Bozorgmanesh et al, 2010	Research Diabetes prediction, lipid accumulation product, and adiposity measures; 6-year follow-up: Tehran lipid and glucose study	lack of data
Bragg et al., 2022A	Circulating Metabolites and the Development of Type 2 Diabetes in Chinese Adults	lack of data
Brahimaj et al., 2017A	Serum Levels of Apolipoproteins and Incident Type 2 Diabetes: A Prospective Cohort Study	not relevant factor
Brahimaj et al., 2017B	Novel metabolic indices and incident type 2 diabetes among women and men: the Rotterdam study	wrong publication type
Cai et al., 2021	Triglyceride to high-density lipoprotein cholesterol ratio variability and incident diabetes: A 7-year prospective study in a Chinese population	not relevant factor
Cardona et al., 2009	El ácido úrico es un predictor de desarrollo de diabetes mellitus tipo 2 en la población general	not relevant factor
Carvalho et al., 2020	Increased particle size of triglyceride remnant lipoproteins, but not plasma concentration or lipid content, boost risk prediction of incident type 2 diabetes	wrong publication type
Carvalho et al., 2020	Increased particle size of triglyceride remnant lipoproteins, but not their plasma concentration or lipid content, augment risk prediction of incident diabetes: prospective results from ELSA-Brasil	wrong publication type
Chang et al., 2005	What will be the proper criteria for impaired fasting glucose for Korean Men?	wrong data
Choi et al., 2009	The association of serum triglyceride changes over 4 years of period with	wrong publication type
Cicero et al., 2005	Relative role of major risk factors for Type 2 diabetes development in the historical cohort of the Brisighella Heart Study: an 8-year follow-up	wrong population
Cui et al., 2017	The association of triglycerides and total cholesterol concentrations with newly diagnosed diabetes in adults in China	wrong study design
Cui et al., 2018	Triglycerides and total cholesterol concentrations in association with IFG/IGT in Chinese adults in Qingdao, China	wrong study design
Cui et al., 2019	The Ability of Baseline Triglycerides and Total Cholesterol Concentrations to Predict Incidence of Type 2 Diabetes Mellitus in Chinese Men and Women: A Longitudinal Study in Qingdao, China	wrong data
Dallmeier et al., 2011	Biomarkers from multiple pathways are not associated with incident type 2 diabetes: The Framingham Heart Study	wrong publication type
de Oliveira et al, 2022	Comparing different metabolic indexes to predict type 2 diabetes mellitus in a five years follow-up cohort: The Baependi Heart Study	lack of data
de Oliveria et al., 2020	Triglyceride glucose index as a tool to motivate early lifestyle modification in young adults at diabetes risk: The Baependi Heart Stud	lack of data
Deberneh et al., 2021	Prediction of type 2 diabetes occurrence using machine learning model	wrong publication type
Demirbas., 2020	Comparison of Triglyceride/Glucose Index with the FINDRISC Diabetes Risk Questionnaire in Determining Diabetes Risk in Individuals Attending Periodic Health Examinations	wrong study design
Diabetes Prevention Program Research Group, 2005	Lipid, Lipoproteins, C-Reactive Protein, and Hemostatic Factors at Baseline in the Diabetes Prevention Progra	wrong study design
Drexel et al., 2017	Visceral adiposity is a significantly stronger predictor of diabetes incidence in men than in women	wrong publication type
Du et al., 2014	Visceral adiposity index, hypertriglyceridemic waist and risk of diabetes: the China Health and Nutrition Survey 2009	wrong study design
Egeland et al., 2011	Hypertriglyceridemic-waist phenotype and glucose intolerance among Canadian Inuit: the International Polar Year Inuit Health Survey for Adults 2007–2008	wrong study design
Fallahzadeh et al., 2019	Population attributable risk of risk factors for type 2 diabetes; Bayesian methods	wrong study design
Feng et al, 2018	Relationship between very low low-density lipoprotein cholesterol concentrations not due to statin therapy and risk of type 2 diabetes: A US-based cross-sectional observational study using electronic health record	wrong study design
Fingeret et al., 2018	Incidence of type 2 diabetes, hypertension, and dyslipidemia in metabolically healthy obese and non-obese	wrong outcome
Fiorentino et al., 2020	HDL cholesterol is an independent predictor of β-cell function decline and incident type 2 diabetes: A longitudinal study	lack of data
Flores-Guerrero et al., 2020	A newly developed diabetes risk index, based on lipoprotein subfractions and branched-chain amino acids, is associated with incident type 2 diabetes mellitus in the prevend cohort	wrong publication type
Gadgil et al., 2021	Circulating metabolites and lipids are associated with glycaemic measures in South Asians	lack of data
Gadgil et al., 2023	Lipoprotein Subfractions and Type 2 Diabetes Incidence in the Mediators of Atherosclerosis in South Asians Living in America (MASALA) and Multiethnic Study of Atherosclerosis (MESA) Cohorts	wrong publication type
Gadgil et al., 2023	Association of lipoprotein subfractions with incidence of type 2 diabetes among five U.S. Race and Ethnic groups: The Mediators of Atherosclerosis in South Asians Living in America (MASALA) and Multi-Ethnic study of Atherosclerosis (MESA)	lack of data
Gang et al., 2016	Association of the Elevated Red Blood Cell Distribution Width with the Risk of Developing Diabetes Mellitu	lack of data
Ganji et al., 2012	Poor vitamin D status is associated with increased risk for metabolic syndrome (MeS), diabetes mellitus (DM), and several unfavorable cardiometabolic markers in US adults	wrong publication type

Gao- et al., 2022	Iron Overload and the Risk of Diabetes in the General Population: Results of the Chinese Health and Nutrition Survey Cohort Study	wrong outcome
Garcia et al., 2019	High Betaine, a Trimethylamine N-Oxide Related Metabolite, Is Prospectively Associated with Low Future Risk of Type 2 Diabetes Mellitus in the PREVEND Study	lack of data
Gasevic et al., 2011	the association between triglyceride to highdensity-lipoprotein ratio and insulin resistance in a multi-ethnic primary prevention cohort	wrong publication type
Gerben Hulsegge et al., 2015	Trajectories of traditional risk factors and novel biomarkers before the onset of type 2 diabetes, the Doetinchem Cohort Stud	wrong publication type
Giannini et al., 2012	Lipid levels in obese and nonobese subjects as predictors of fasting and postload glucose metabolism	wrong study design
Goedecke etal., 2022	Waist circumference thresholds predicting incident dysglycaemia and type 2 diabetes in Black African men and wome	lack of data
Hällfors et al., 2019	High-throughput metabolomics for early detection of individuals at increased risk for type 2 diabetes	wrong publication type
Han et al., 2016	Incidence and predictors of type 2 diabetes mellitus among Koreans: A 12- year follow up of the Korean Genome and Epidemiology Study	wrong population
Han et al., 2023	Triglyceride glucose-body mass index and the risk of progression to diabetes from prediabetes: A 5-year cohort study in Chinese adults	wrong population
Hanson et al., 2017	Serum lipids and incidence of diabetes in an American-Indian population	wrong publication type
He et al., 2023	Association of four lipid-derived indicators with the risk of developing type 2 diabetes: a Chinese population-based cohort stud	lack of data
Heianza et al., 2012	type 2 diabetes risk in Korean adults	wrong publication type
Henninger et al., 2014	Metabolic and Adipose Tissue Predictors of Impaired Glucose Tolerance and Type 2 Diabetes—A Prospective Cohort Study	wrong publication type
Hjellvik et al., 2012	Body mass index, triglycerides, glucose, and blood pressure as predictors of type 2 diabetes in a middle-aged Norwegian cohort of men and women	wrong outcome
Hu et al., 2009	Association of Serum C-Reactive Protein Level with Sex-Specific Type 2 Diabetes Risk: A Prospective Finnish Stud	lack of data
Huang et al., 2020	Triglycerides, uric acid, and incidence of type 2 diabetes mellitus in northwest china: the jinchang cohort study	wrong publication type
Hung et al., 2021	Non-skilled occupation as a risk factor of diabetes among working population: A population-based study of communitydwelling adults in Hong Kong	wrong study design
Husseini et al., 2003	Selected factors associated with diabetes mellitus CR in a rural Palestinian communi	wrong study design
Hwang et al., 2012	Circulating Osteocalcin Level Is Not Associated With Incident Type 2 Diabetes in Middle-Aged Male Subject	lack of data
Imamura et al., 2012	Risk factors for type 2 diabetes mellitus preceded by β-cell dysfunction, insulin resistance, or both: The cardiovascular health study	wrong publication type
Janghorbani et al., 2016B	The Visceral Adiposity Index in Comparison with Easily Measurable Anthropometric Markers Did Not Improve Prediction of Diabetes	not relevant factor
Jiang et al., 2021	Triglyceride glucose-body mass index in identifying high-risk groups of pre-diabetes	not relevant factor
Jing et al., 2023	Increased TG Levels and HOMA-IR Score Are Associated With a High Risk of Prediabetes: A Prospective Study	lack of data
Johnston et al., 2016	Triglyceride Fatty Acid (TGFA) Composition Longitudinally Associates with Changes in Insulin Sensitivity (IS) and β-Cell Function Over 6 Years in the Prospective Metabolism and Islet Cell Evaluation (PROMISE) Coho	wrong publication type
Johnston et al., 2018	Clusters of fatty acids in the serum triacylglyceride fraction associate with the disorders of type 2 diabete	lack of data
Kahn et al., 2009	Two Risk-Scoring Systems for Predicting Incident Diabetes Mellitus in U.S. Adults Age 45 to 64 Year	lack of data
Kayikçioğlu et al., 2020	Uzun dönem fibrat tedavisi alan hipertrigliseridemi hastalarinda kümülatif HDL-disi kolesterol yükü: Bir lipit klinik kohortunun gerçek hayat verileri	wrong population
Ke et al., 2022	Comparison of obesity indices and triglyceride glucose-related parameters to predict type 2 diabetes mellitus among normal-weight elderly in China	wrong study design
Kim H. et al., 2021	Prognostic Value of Triglyceride and Glucose Index for Incident Type 2 Diabetes beyond Metabolic Health and Obesity	not relevant factor
Kistorp et al., 2011	The Predictive Ability of Retinol-Binding Protein 4 and High Molecular Weight Adiponectin with Respect to Incident Type 2 Diabetes Mellitus in the General Population	wrong publication type
Klimentidis et al., 2015	Triglyceride-Increasing Alleles Associated with Protection against Type-2 Diabetes	lack of data
Knekt et al., 2011	Serum Triglyceride, related to an elevated type 2 diabetes risk, predict a reduced risk of Parkinson's disease	wrong publication type
Koohi et al., 2022	Patterns of change in obesity indices and other cardiometabolic risk factors before the diagnosis of type 2 diabetes: two decades follow-up of the Tehran lipid and glucose study	wrong outcome
Kuang et al., 2022	Association of LDL:HDL ratio with prediabetes risk: a longitudinal observational study based on Chinese adult	not relevant factor
Lam et al., 2016	Obesity indices vs. 2-hour plasma glucose for predicting incident diabetes in a 16-year prospective study of Hong Kong Chinese	wrong publication type

Lan et al., 2023	Temporal relationship between atherogenic dyslipidemia and infammation and their joint cumulative efect on type 2 diabetes onset: a longitudinal cohort study	lack of data
Lee B. et al., 2016	Identification of Type 2 Diabetes Risk Factors Using Phenotypes Consisting of Anthropometry and Triglycerides based on Machine Learning	wrong study design
Lee et al., 2015	Changes in Metabolic Health Status Over Time and Risk of Developing Type 2 Diabetes	not relevant factor
Lee et al., 2018	The number of metabolic syndrome components and incidence rates of type 2 diabetes-a Korean national sample cohort study	wrong publication type
Lee et al., 2020A	Changes in metabolic syndrome and its components and the risk of type 2 diabetes: a nationwide cohort study	not relevant factor
Leong et al., 2017	Type 2 diabetes (T2D) prediction by hemoglobin A1C in real-world scenarios	wrong publication type
Leong et al., 2018	Prediction of Type 2 Diabetes by Hemoglobin A1c in Two Community-Based Cohort	lack of data
Ley et al., 2011	Prognostic utility of non-high density lipoprotein (HDL) cholesterol in assessing type 2 diabetes risk	wrong publication type
Li et al., 2015	Prevalence of diabetes mellitus and impaired fasting glucose, Associated with risk factors in rural Kazakh adults in Xinjiang, China	wrong study design
Li et al., 2017	A point-based prediction model for predicting 10-year risk of developing type 2 diabetes mellitus in Japanese men: Aichi workers' cohort study	wrong publication type
Li et al., 2021.2	Correction to: Association between triglyceride-glucose index and risk of incident diabetes: a secondary analysis based on a Chinese cohort stud	wrong publication type
Li et al., 2022	Predictive Effect of Triglyceride Glucose−Related Parameters, Obesity Indices, and Lipid Ratios for Diabetes in a Chinese Population: A Prospective Cohort Study	lack of data
Liberty et al., 2021	Triglyceride/Glucose Index (TyG Index) as A Marker of Glucose Status Conversion among Reproductive-Aged Women in Jakarta, Indonesia: The Bogor Cohort Study (2011-2016)	wrong data
Liberty et al., 2023	Predictors of Prediabetes Among Communities Without a Family History of Type 2 Diabetes Mellitus: A Case-Control Study	wrong study design
Lieb et al., 2022	Clinical correlates of plasma insulin levels over the life course and association with incident type 2 diabetes: the Framingham Heart Study	wrong outcome
Lin et al., 2014	Incidence and risk factors of diabetes among Chinese subjects aged over 55 years: A community-based study	wrong publication type
Lin et al., 2021	Prediction of metabolic syndrome with impaired glucose tolerance in type 2 diabetes mellitus: a cohort study	wrong population
Lindquist et al., 2009	Predictors of diabetes type II in normoglycemic and prediabetic subjects	wrong publication type
L'Italien et al., 2009	Triglyceride to HDL-C ratio as predictor of new onset diabetes in general populations; A longitudinal database analysis	wrong publication type
Liu et al., 2017A	Metabolomics based markers predict type 2 diabetes in a 14-year follow-up stud	lack of data
Liu et al., 2017B	Different contributions of lipid profiles and BMI to the natural history of type 2 diabetes: A 3-year cohort study in China	wrong publication type
Liu et al., 2020	Association between Triglyceride-Glucose Index and Type 2 Diabetes Mellitus in the Japanese Population: A Secondary Analysis of a Retrospective Cohort Stud	lack of data
Luft et al., 2013	Retinol binding protein 4 and incident diabetes – the Atherosclerosis Risk in Communities Study (ARIC Study)	lack of data
Mader et al., 2018	Visceral adiposity is a significantly stronger predictor of diabetes incidence in men than in women	wrong publication type
Mainous et al., 2007	Assessing Risk for Development of Diabetes in Young Adult	lack of data
Malmström et al., 2017	Subtle elevations of metabolic risk factors prognosticate type 2 diabetes 20 years or more before diagnosis-a study with 47,997 cases	wrong publication type
Mann et al., 2009	Comparative validity of 3 diabetes risk prediction scoring methods in a multi-ethnic cohort: The multi-ethnic study of atherosclerosis (MESA)	wrong publication type
Marjara et al., 2022	Natriuretic Peptide Levels and Predicting Risk of Developing New Diabetes Mellitus or Metabolic Syndrome: A Population Based Study	wrong publication type
Marques-Vidal et al., 2012	Adipocytokines, Hepatic and Inflammatory Biomarkers and Incidence of Type 2 Diabetes. The CoLaus Study	lack of data
Maskarinec 2017	A priori-defined diet quality indexes, biomarkers, and risk of type 2 diabetes in five ethnic groups: The Multiethnic Cohort	wrong publication type
Maskarinec et al., 2018	Sleep Duration and Incidence of Type 2 Diabetes: The Multiethnic Cohor	lack of data
Maturu et al., 2015	The Triglyceride to High-Density Lipoprotein Cholesterol (TG/HDL-C) Ratio as a Predictor of β-Cell Function in African American Wom	wrong population
Mehrabi et al., 2010	Prediction of diabetes using logic regression	lack of data
Mellor et al., 2020	Association between lipids and apolipoproteins on type 2 diabetes risk; moderating effects of gender and polymorphisms; the ATTICA study	lack of data
Methot et al., 2009	Relation of the “hypertriglyceridemic waist” phenotype to glucose intolerance and type 2 diabetes in postmenopausal women	wrong publication type
Miljkovic et al., 2019	Incidence and risk factors for type 2 diabetes among the offspring of long-lived individuals: the long life family study	wrong publication type
Miyakoshi et al., 2016A	Development of new diabetes risk scores on the basis of the current definition of diabetes in Japanese subject	lack of data
Miyakoshi et al., 2016B	Favorable performance of noninvasive diabetes risk score for the diabetes diagnosed by the current criteria	wrong publication type

Møller et al., 2011	Serum Soluble CD163 Predicts Risk of Type 2 Diabetes in the General Population	lack of data
Moro et al., 2003	Hypertriglyceridemia Is Associated With Increased Insulin Resistance in Subjects With Normal Glucose Tolerance: Evaluation in a Large Cohort of Subjects Assessed With the 1999 World Health Organization Criteria for the Classification of Diabetes	wrong study design
Mueller et al., 2014	Earlier age at menarche is associated with higher diabetes risk and cardiometabolic disease risk factors in Brazilian adults: Brazilian Longitudinal Study of Adult Health (ELSA-Brasil	lack of data
Muhammad et al., 2022	Triglyceride-glucose (TyG) index is a predictor of arterial stiffness, incidence of diabetes, cardiovascular disease, and all-cause and cardiovascular mortality: A longitudinal two-cohort analysis	lack of data
Muniz et al., 2022	Diabetes mellitus prediction based on the triglyceride and glucose index	lack of data
Nair et al., 2021	Associations of Body Mass Index and Waist Circumference in Young Adulthood with Later Life Incident Diabete	lack of data
Namayandeh et al., 2019	The incidence rate of diabetes mellitus (type II) and its related risk factors: A 10-year longitudinal study of Yazd Healthy Heart Cohort (YHHC), Iran	lack of data
Nasrallah et al., 2022	Incidence of diabetes and its predictors in the Greater Beirut Area: a fve-year longitudinal study	lack of data
Navarro-Gonzále et al., 2016A	TyG Index Change Is More Determinant for Forecasting Type 2 Diabetes Onset Than Weight Gain	lack of data
Navarro-Gonzále et al., 2016B	TYG index may be useful for the early identification of patients at risk of type 2 diabetes	wrong publication type
Nazmul ahasan et al., 2011	Prevalence and Risk Factors of Type 2 Diabetes Mellitus Among Secretariat Employees of Bangladesh	wrong study design
Nguyen et al., 2012	Correlates of age-onset of type 2 diabetes mellitus among relatively young black and white adults in a community: The bogalusa heart study	wrong publication type
Nguyen et al., 2012	Correlates of Age Onset of Type 2 Diabetes Among Relatively Young Black and White Adults in a Communit	wrong population
Nichols et al., 2010	The risk of incident diabetes for all possible combinations of metabolic syndrome components	wrong publication type
Njeru et al., 2012	Assessment of type 2 diabetes risk factors available in the electronic medical record by using association rule mining	wrong publication type
Onat et al., 2011	Serum γ-Glutamyltransferase: Independent Predictor of Risk of Diabetes, Hypertension, Metabolic Syndrome, and Coronary Disease	wrong outcome
Onat et al., 2013	Fasting, non-fasting glucose and HDL dysfunction in risk of pre-diabetes, diabetes, and coronary disease in non-diabetic adults	lack of data
Oresic et al., 2015	Serum lipidome as an independent predictor of progression to type 2 diabetes: the METSIM study	wrong publication type
Ouni et al., 2019	Insulin-like growth factor binding protein 2 (IGFBP-2) and the risk of developing type 2 diabetes	wrong publication type
Owei et al., 2016A	Plasma Lipid Profi le, Insulin Action, and Prediabetes Risk in Healthy Black and White Offspring of Parents with Type 2 Diabetes	wrong publication type
Owei et al., 2017	Insulin-sensitive and insulin-resistant obese and non-obese phenotypes: role in prediction of incident pre-diabetes in a longitudinal biracial cohort	lack of data
Pan et al., 2022	Comparison of longitudinal changes in four surrogate insulin resistance indexes for incident T2DM in middle-aged and elderly Chinese	lack of data
Pang et al., 2018	[Association of lipid parameters with insulin resistance in different glycemic among the elderly population]	wrong publication type
Pankow et al., 2004	Fasting Plasma Free Fatty Acids and Risk of Type 2 Diabetes	lack of data
Paolisso et al., 1995A	A high concentration of fasting plasma non-esterified fatty acids is a risk factor for the development of NIDDM	lack of data
Paolisso et al., 1995B	A high concentration of fasting plasma non-esterified fatty acids is a risk factor for the development of NIDDM	lack of data
Parcha et al., 2022	Genetic European Ancestry and Incident Diabetes in Black Individuals: Insights from the SPRINT Trial	wrong study design
Qi et al., 2011	Genetic predisposition to dyslipidemia and type 2 diabetes risk in two prospective cohorts	wrong publication type
Qi et al., 2012	Genetic Predisposition to Dyslipidemia and Type 2 Diabetes Risk in Two Prospective Cohort	lack of data
Ram et al., 2014	Hypertriglyceridaemic waist phenotype as a simple predictive marker of incident diabetes in Asian-Indian men with prediabetes	wrong population
Ramezankhani et al., 2014	Applying decision tree for identification of a low risk population for type 2 diabetes. Tehran Lipid and Glucose Study	lack of data
Ramezankhani et al., 2019	Sex differences in the association between spousal metabolic risk factors with incidence of type 2 diabetes: a longitudinal study of the Iranian population	wrong population
Ramírez-Vélez et al., 2019	Obesity- and Lipid-Related Parameters in the Identification of Older Adults with a High Risk of Prediabetes According to the American Diabetes Association: An Analysis of the 2015 Health, Well-Being, and Aging Study	wrong study design
Rhee et al., 2014	Metabolic Health Is a More Important Determinant for Diabetes Development than Simple Obesity: A 4-Year Retrospective Longitudinal Study	wrong study design
Rhee et al., 2015	Increased risk of diabetes development in subjects with the hypertriglyceridemic waist phenotype: A 4-year longitudinal study	wrong publication type

Saely et al., 2018	The visceral adiposity index is a significantly stronger predictor of incident diabetes in men than in wome	wrong publication type
Salomaa et al., 2009	Thirty novel biomarkers as predictors of clinically incident diabetes	wrong publication type
Sánchez-Iñigo et al., 2016	Metabolic health categories and risk of developing type 2 diabetes	wrong publication type
Sane et al., 1993	Does Familial Hypertriglyceridemia Predispose to NIDDM	lack of data
Santos-Ferreira et al., 2019	Apolipoprotein E2 Genotype Is Associated with a 2-Fold Increase in the Incidence of Type 2 Diabetes Mellitus: Results from a Long-Term Observational Study	lack of data
Sato et al., 2013	Serum butyrylcholinesterase and the risk of future type 2 diabetes: The Kansai Healthcare Study Short title: butyrylcholinesterase and type 2 diabetes	lack of data
Schaefer et al., 2019	Diabetes prediction and prevention	wrong publication type
Schaefer et al., 2023	The Prediction and Prevention of Diabetes Mellitus	wrong publication type
Schmid et al., 2012	Validation of 7 type 2 diabetes mellitus risk scores in a population-based cohort: CoLaus study	wrong publication type
Schmidt et al., 2005	Identifying Individuals at High Risk for Diabetes	lack of data
Schmidt et al., 2006	Leptin and incident type 2 diabetes: risk or protection	lack of data
Schneider et al., 2012	Boehringer Ingelheim employee study: A prospective epidemiological cohort study diabetes prevalence and association between impaired glucose metabolism and risk factors	wrong publication type
Schulze et al., 2008	Validierung des Deutschen Diabetes-Risiko-Scores mit metabolischen Risikofaktoren für Typ-2-Diabetes Validation of the German Diabetes Risk Score with metabolic risk factors for type 2 diabete	Lack of data
Schulze et al., 2009	Use of Multiple Metabolic and Genetic Markers to Improve the Prediction of Type 2 Diabetes: the EPIC-Potsdam Stud	lack of data
Schulze et al., 2010	Fasting plasma glucose and Type 2 diabetes risk: a non-linear relationship	lack of data
Seah et al., 2028	Risk prediction models for type 2 diabetes using either fasting plasma glucose or HbA1c in Chinese, Malay, and Indians: Results from three multi-ethnic Singapore cohorts	lack of data
Sen et al., 2010	High triglycerides may predict the new onset of type 2 diabetes in nondiabetic patients: A 15-year prospective study in a general population	wrong publication type
Seo et al., 2023	Lipid Profile and Visit-to-Visit Variability of LDL Cholesterol, the Association with Risk of Young-Onset Type 2 Diabetes- Nationwide Retrospective Cohort Study	wrong publication type
Serrano-Rios et al., 2011	Ability of hemoglobin A1C to detect individuals at increased risk of type 2 diabetes as compared with that of fasting and 2-H glucose: The segovia primary care study (SPCS)	wrong publication type
Shafie et al., 2009	Comparison of waist-to-height ratio and body mass index for prediction of type 2 diabetes mellitus risk in women: Tehran lipid and glucose study	not relevant factor
Shang et al., 2013A	Educational level, obesity and incident diabetes among Chinese adult men and women aged 18-59 years old: An 11-Year follow-up Study	wrong publication type
Shang et al., 2013B	Educational Level, Obesity and Incidence of Diabetes among Chinese Adult Men and Women Aged 18–59 Years Old: An 11-Year Follow-Up Study	lack of data
Shao et al., 2018	Hypertriglyceridemia is identified as a risk factor of hyperglycemia via bayesian network inference	wrong publication type
Sharafi et al., 2023	Predictive value of triglycerides to high-density lipoprotein cholesterol and triglyceride glycemic index for diabetes incidence in pre-diabetes patients: a prospective cohort stud	wrong population
Sheu et al., 2006	Predictors of incident diabetes, metabolic syndrome in middle-aged adults: A 10-year follow-up study from Kinmen, Taiwa	wrong data
Shi et al., 2018	Estimate of prevalent diabetes from cardiometabolic index in general Chinese population: a community-based stud	wrong study design
Shimodaira et al., 2014	Impact of Serum Triglyceride and High Density Lipoprotein Cholesterol Levels on Early-Phase Insulin Secretion in Normoglycemic and Prediabetic Subject	wrong study design
Shrestha et al., 2022	Prevalence of diabetes mellitus and associated risk factors in Nepal: findings from a nationwide populationbased surve	wrong study design
Shrivastava et al., 2014	High prevalence of type 2 diabetes melitus and its risk factors among the rural population of Pondicherry, South India	wrong study design
Simental-Mendína et al., 2008	The Product of Fasting Glucose and Triglycerides As Surrogate for Identifying Insulin Resistance in Apparently Healthy Subjects	wrong study design
Simental-Mendína et al., 2015	The hypertriglyceridemia is associated with isolated impaired glucose tolerance in subjects without insulin resistanc	wrong study design
Skarn et al., 2015	Family history of hypertension and serum triglycerides predict future insulin sensitivity: a 17-year follow-up study of young men	lack of data
Skoumas et al., 2023	Lipoprotein(a), metabolic profile and new-onset type 2 diabetes in patients with familial combined hyperlipidemia: A 9 year follow-up study	not relevant factor
Snehalatha et al., 2005	Serum triglycerides as a marker of insulin resistance in non-diabetic urban Indian	wrong publication type
Snijder et al., 2016	Associations of Adiponectin Levels With Incident Impaired Glucose Metabolism and Type 2 Diabetes in Older Men and Women	lack of data
Sokooti et al., 2021	Triglyceride-rich lipoprotein and LDL particle subfractions and their association with incident type 2 diabetes: the PREVEND study	not relevant factor
Song et al., 2015	Performance of HbA1c for the prediction of diabetes in a rural community in Kore	not relevant factor
Song et al., 2016A	Gender-related affecting factors of prediabetes on its 10-year outcom	wrong population
Song et al., 2016B	Associations between non-traditional lipid measures and risk for type 2 diabetes mellitus in a Chinese community population: a cross-sectional study	wrong study design

Song et al., 2022	Triglyceride Glucose-Body Mass Index and Risk of Incident Type 2 Diabetes Mellitus in Japanese People With Normal Glycemic Level: A Population-Based Longitudinal Cohort Study	not relevant factor
Soremekun et al., 2022	Lipid traits and type 2 diabetes risk in African ancestry individuals: A Mendelian Randomization study	wrong population
Sotoodeh Jahromi et al., 2015	Prevalence of prediabetes and its association to cardiovascular risk factors	wrong publication type
Stefan et al., 2008	Plasma Fetuin-A Levels and the Risk of Type 2 Diabetes	lack of data
Stern et al., 2002	Identification of Persons at High Risk for Type 2 Diabetes Mellitus: Do We Need the Oral Glucose Tolerance Test	lack of data
Stern et al., 2004	Does the Metabolic Syndrome Improve Identification of Individuals at Risk of Type 2 Diab	lack of data
Stringhini et al., 2012B	Contribution of modifiable risk factors to social inequalities in type 2 diabetes incidence. The British Whitehall II Study	wrong publication type
Sujana et al., 2020	Associations of cardiac stress biomarkers with incident type 2 diabetes and changes in glucose metabolism: KORA F4/FF4 study	not relevant factor
Sumner et al., 2008	Ethnic differences in the ability of triglyceride levels to identify insulin resistance	wrong study design
Sumner et al., 2010	The Triglyceride/High-Density Lipoprotein Cholesterol Ratio Fails to Predict Insulin Resistance in African-American Women: An Analysis of Jackson Heart Study	wrong study design
Sun et al., 2011	The association of gamma-glutamyltransferase and C-reactive protein with IFG/IGT in Chinese adults in Qingdao, Chin	wrong study design
Szili-Torok et al., 2022A	Fasting ketone bodies and incident type 2 diabetes in the general population	wrong publication type
Tabák et al., 2015	Incremental value of a past fasting glucose for the prediction of type 2 diabetes mellitus. The Whitehall II study	wrong publication type
Talmud et al., 2010	Utility of genetic and non-genetic risk factors in prediction of type 2 diabetes: Whitehall II prospective cohort study	lack of data
Tao et al., 2016	Longitudinal Associations between Triglycerides and Metabolic Syndrome Components in a Beijing Adult Population, 2007-2012	lack of data
Taylor et al., 2009	Diabetes incidence: Seven-fold risk among those with metabolic syndrome	wrong publication type
Thanikachalam et al., 2013	Gender-specific plasma triglyceride/high-density lipoprotein cholesterol concentration ratio levels to identify insulin resistance and associated cardiometabolic risk factors in South Asian population	wrong publication type
Tikkaenem et al., 2019	State of the art metabolomics enables early detection, risk stratification and personalized follow-up in patients at increased risk for type 2 diabetes: study in 11896 young adults	wrong publication type
Tirosh et al., 2005	Normal Fasting Plasma Glucose Levels and Type 2 Diabetes in Young Men	not relevant factor
Tohidi et al., 2013	Non-linear association between 25-hydroxyvitamin D and the incidence of Type 2 diabetes: a community-based nested case–control study	not relevant factor
Tricò et al., 2018	Triglyceride-rich very low density lipoproteins (VLDL) are independently associated with insulin secretion in a multiethnic cohort of adolescents	wrong population
Tsirona et al., 2016	Prevalence and determinants of type 2 diabetes mellitus in a Greek adult populatio	wrong study design
Tsur et al., 2013	Decreased Serum Concentrations of 25-Hydroxycholecalciferol Are Associated With Increased Risk of Progression to Impaired Fasting Glucose and Diabetes	lack of data
Tulloch-Reid et al., 2010	Appropriate waist circumference cut points for identifying insulin resistance in black youth: A cross sectional analysis of the 1986 Jamaica birth cohort	wrong study design
Twig et al., 2013	White Blood Cells Count and Incidence of Type 2 Diabetes in Young Men	lack of data
Valdivielso et al., 2009	Association of moderate and severe hypertriglyceridemia with obesity, diabetes mellitus and vascular disease in the Spanish working population: Results of the ICARIA study	wrong study design
Vanderpump et al., 1996	The incidence of diabetes mellitus in an English community: a 20-year follow-up of the Whickham Survey	wrong publication type
Vanderpump et al., 1996	The Incidence of Diabetes Mellitus in an English Community: A 20-year Follow-up of the Whickham Surve	lack of data
Vasan et al., 2012	Fasting blood glucose predicts the risk of developing type 2 diabetes over 15 years among rural south indians	wrong publication type
Vatcheva et al., 2019	Sex and Age Differences in Prevalence and Risk Factors for Prediabetes in MexicanAmericans	wrong study design
Vega et al., 2015	Diabetes and obesity incidence in 5 years follow up of a Spanish cohort	wrong publication type
von Bibra et al., 2017	Impact of the Triglyceride/High-Density Lipoprotein Cholesterol Ratio and the Hypertriglyceremic-Waist Phenotype to Predict the Metabolic Syndrome and Insulin Resistance	wrong study design
Vonbank et al., 2017	Visceral adiposity is a significantly stronger predictor of diabetes incidence in men than in women	wrong publication type
Vonbank et al., 2017	Visceral adiposity is a significantly stronger predictor of diabetes incidence in men than in women	wrong publication type
Vonbank et al., 2018	Visceral adiposity is a significantly stronger predictor of diabetes incidence in men than in women	wrong publication type
Wang et al., 2004	The Metabolic Syndrome Defined by Factor Analysis and Incident Type 2 Diabetes in a Chinese Population With High Postprandial Glucos	lack of data
Wang et al., 2016	Risk scores for predicting incidence of type 2 diabetes in the Chinese population: the Kailuan prospective study	lack of data

Wang et al., 2020	Multiple Biomarkers Improved Prediction for the Risk of Type 2 Diabetes Mellitus in Singapore Chinese Men and Women	lack of data
Wang et al., 2021B	Visceral adiposity is a significantly stronger predictor of diabetes incidence in men than in women	wrong publication type
Wang et al., 2021C	Triglyceride glucose-body mass index and the risk of diabetes: a general populationbased cohort study	not relevant factor
Wang et al., 2022	Visit-to-visit variability in triglyceride-glucose index and diabetes: A 9-year prospective study in the Kailuan Study	not relevant factor
Watanabe et al., 2016	Risk of Future Diabetes in Japanese People with High-normal Fasting Plasma Glucose Levels: A 4-Year Follow-up Study	wrong population
Wei et al., 2011A	Blood pressure and the risk of developing diabetes in African Americans and Whites: ARIC, CARDIA, and the Framingham Heart Study	wrong publication type
Wei et al., 2011B	Blood Pressure and the Risk of Developing Diabetes in African Americans and Whites	lack of data
Wei et al., 2012	Uric acid is a predictor of type 2 diabetes, hypertension, and liver function in a Chinese senior cohort	wrong publication type
Welsh et al., 2011	NT-proBNP and risk of incident Type 2 diabetes: Results from a prospective study in WOSCOPS	wrong publication type
Wen et al., 2020	Elevated triglyceride-glucose (TyG) index predicts incidence of Prediabetes: a prospective cohort study in China	lack of data
Wenije et al., 2015	Prospective association of fatty acids in the de novo lipogenesis pathway with risk of type 2 diabetes: the Cardiovascular Health Study1	lack of data
Wilkinson et al., 2020	Development and validation of a model for predicting incident type 2 diabetes using quantitative clinical data and a Bayesian logistic model: A nationwide cohort and modeling study	lack of data
Wilkinson et al., 2023	Correction:Development and validation of a model for predicting incident type 2 diabetes using quantitative clinical data and a Bayesian logistic model: A nationwide cohort and modeling study	wrong publication type
Wilson et al., 2011	Type 2 Diabetes Risk in Persons with Dysglycemia: The Framingham Offspring Study	wrong population
Wu et al., 2021	Applying latent class analysis to risk stratification of incident diabetes among Chinese adults	lack of data
Würtz et al., 2019	Circulating metabolites and the risk of type 2 diabetes: a prospective study of 11,896 young adults from four Finnish cohorts	wrong publication type
Xia et al., 2013	Prevalence and Risk Factors of Type 2 Diabetes in the Adults in Haikou City, Hainan Island, China	wrong study design
Xuan et al., 2022	Impacts of Triglyceride Glucose-Waist to Height Ratio on Diabetes Incidence: A Secondary Analysis of A Population-Based Longitudinal Data	lack of data
Yang et al., 2009	Markers of liver dysfunction and risk of type 2 diabetes in chinese adult population	wrong publication type
Yang et al., 2018	Serum metabolite profle associated with incident type 2 diabetes in Koreans: fndings from the Korean Genome and Epidemiology Stud	lack of data
Yang et al., 2022	Lipid Accumulation Product Index Predicts New-Onset Type 2 Diabetes Among Non-Obese Koreans: A 12-Year Longitudinal Study	lack of data
Yazdani et al., 2013	Non-linear association of vitamin d and incident type 2 diabetes; results of a nested case-control study among Amiddle east population	wrong publication type
Yeh et al., 2005	Vital Capacity as a Predictor of Incident Type 2 Diabete	lack of data
Yetukuri et al., 2013	Serum lipidomic profiling identifies biomarkers associated with progression to type 2 diabetes in the METSIM study	wrong publication type
Yi et al., 2005	Analysis of risk factors on type 2 diabetes mellitus in some communities of Urumchi	wrong publication type
Yi et al., 2022	The longitudinal efect of the atherogenic index of plasma on type 2 diabetes in middle-aged and older Chines	lack of data
Yoon et al., 2022	Lipid Accumulation Product Predicts New-onset Type 2 Diabetes Among Lean Koreans	wrong publication type
Young et al., 1994	Risk of clinical diabetes in a Northern Native Canadian cohort	lack of data
Yu et al., 2016	Study and Evaluation the Impact of VDR Variants on the Risk of T2DM in Han Chines	wrong study design
Yu et al., 2021	Transition of Lipid Accumulation Product Status and the Risk of Type 2 Diabetes Mellitus in Middle-Aged and Older Chinese: A National Cohort Study	not relevant factor
Yu et al., 2021b	Transition of Lipid Accumulation Product Status and the Risk of Type 2 Diabetes Mellitus in Middle-Aged and Older Chinese: A National Cohort Study	not relevant factor
Yu et al., 2022	Temporal sequence of blood lipids and insulin resistance in perimenopausal women: the study of women's health across the nation	lack of data
Zaccardi et al., 2015	Serum fructosamine and risk of type 2 diabetes mellitus among middle-age Finnish men: a 23-year population-based prospective study	not relevant factor
Zaccardi et al., 2015A	Serum fructosamine and risk of type 2 diabetes mellitus among middle-age Finnish men: a 23-year population-based prospective study	wrong publication type
Zaccardi et al., 2015B	Association between direct measurement of active serum calcium and risk of type 2 diabetes mellitus: A prospective study	not relevant factor
Zar et al., 2022	Visceral AdiposityIndex: A simple tool for assessing risk of type 2 diabetes mellitus	wrong study design
Zha et al., 2023	The nonlinear correlation between the cardiometabolic index and the risk of diabetes: A retrospective Japanese cohort study	not relevant factor
Zhang et al., 2009	Blood lipid levels in relation to glucose status in seven populations of Asian origin without a prior history of diabetes: the DECODA study	wrong study design

Zhang et al., 2017	Baseline TG/HDL predicts future type 2 diabetes in a 12year follow up of Mexican Americans in Starr County, TX: 20022014	wrong publication type
Zhang et al., 2019	Sex-Based Differences in Diabetes Prevalence and Risk Factors: A Population-Based Cross-Sectional Study Among Low-Income Adults in China	wrong study design
Zhang et al., 2020	Association of TG/HDLC ratio trajectory and risk of type 2 diabetes: A retrospective cohort study in China	not relevant factor
Zhang et al., 2020A	TXNIP hypomethylation and its interaction with obesity and hypertriglyceridemia increase type 2 diabetes mellitus risk: A nested case-control study	not relevant factor
Zhang et al., 2021	Association of TG/HDLC ratio trajectory and risk of type 2 diabetes: A retrospective cohort study in China Running title: TG/HDLC ratio trajectory and T2DM	lack of data
Zhang et al., 2022	Comparison of the predictive power of adiposity indices and blood lipid indices for diagnosis of prediabetes	wrong study design
Zhao et al., 2015	Association of low levels of HDL-C or ApoA1 with development of type 2 diabetes mellitus	wrong publication type
Zhao et al., 2016A	Prevalence of Pre-Diabetes and Its Associated Risk Factors in Rural Areas of Ningbo, Chin	wrong study design
Zhao et al., 2016B	Prevalence of Pre-Diabetes and Its Associated Risk Factors in Rural Areas of Ningbo, China	wrong study design
Zhao et al., 2019B	The prevalence and associated factors of type 2 diabetes in rural areas of Ningbo, China	wrong study design
Zhao et al., 2020A	A Linear Relationship Between a Body Shape Index and Risk of Incident Type 2 Diabetes: A Secondary Analysis Based on a Retrospective Cohort Study in Japan	lack of data
Zhao et al., 2020B	The Dose-Response Relationship between Gamma-Glutamyl Transferase and Risk of Diabetes Mellitus Using Publicly Available Data: A Longitudinal Study in Japa	lack of data
Zhao et al., 2022	Association between triglyceride, high-density lipoprotein cholesterol and type 2 diabetes mellitus: A prospective cohort study	lack of data
Zheng et al., 2016	Triglyceride glucose-waist circumference, a novel and effective predictor of diabetes in first-degree relatives of type 2 diabetes patients: cross-sectional and prospective cohort study	wrong population
Zheng et al., 2022	Lower handgrip strength levels probably precede triglyceride glucose index and associated with diabetes in men not in women	not relevant factor
Zheng et al., 2023	Nonlinear relationship between atherogenic index of plasma and the risk of prediabetes: a retrospective study based on Chinese adult	not relevant factor
Zhong et al., 2022	Specifc triacylglycerol, diacylglycerol, and lyso-phosphatidylcholine species for the prediction of type 2 diabetes: a ~16-year prospective study in Chines	not relevant factor
Zhu et al., 2020	Novel plasma lipids predict risk of diabetes: A longitudinal lipidomics study in american indians	wrong publication type
Zou et al., 2017	Analysis of risk factors and their interactions in type 2 diabetes mellitus: A cross-sectional survey in Guilin, Chin	wrong study design
**Studies exluded at full-text screening stage (Identification via Reference Chaser) n=31**		
**First author and year of publication**	**Title**	**Reason**
Achilike et al., 2014	Predicting the development of the metabolically healthy obese phenotype	wrong outcome
Ahn et al., 2019	Visceral adiposity index (VAI), lipid accumulation product (LAP), and product of triglycerides and glucose (TyG) to discriminate prediabetes and diabetes	not relevant factor
Azarpazhooh et al., 2021	Triglyceride/High-Density Lipoprotein Cholesterol Ratio: A Clue to Metabolic Syndrome, Insulin Resistance, and Severe Atherosclerosis	wrong study design
Bovet et al., 2006	The Prediction of Insulin Resistance With Serum Triglyceride and High-Density Lipoprotein Cholesterol Levels in an East African Population	wrong publication type
Charles et al., 1991	Risk Factors for NIDDM in White Population	lack of data
De Silva et al., 2011	Mendelian Randomization Studies Do Not Support a Role for Raised Circulating Triglyceride Levels Influencing Type 2 Diabetes, Glucose Levels, or Insulin Resistance	wrong study design
Gasevic et al., 2012	The association between triglyceride to high-density- lipoprotein cholesterol ratio and insulin resistance in a multiethnic primary prevention cohort	wrong study design
Han et al., 2020	Incidence and risk factors of type 2 diabetes mellitus in individuals with different fasting plasma glucose levels	lack of data
Harati et al., 2009	Impaired fasting glucose cutoff value of 5.6 mmol/l combined with other cardiovascular risk markers is a better predictor for incident Type 2 diabetes than the 6.1 mmol/l value: Tehran lipid and glucose study	lack of data
Kato et al., 2009	Fasting Plasma Glucose and Incidence of Diabetes --- Implication for the Threshold for Impaired Fasting Glucose: Results from the Population-Based Omiya MA Cohort Study	lack of data
Kim-Dorner et al., 2010	Should triglycerides and the triglycerides to high-density lipoprotein cholesterol ratio be used as surrogates for insulin resistance?	wrong study design
Kwon et al., 2018	The Association between Persistent Hypertriglyceridemia and the Risk of Diabetes Development: The Kangbuk Samsung Health Study	lack of data
Li et al., 2014	Are hypertriglyceridemia and low HDL causal factors in the development of insulin resistance?	wrong study design
Lin et al., 2019	A nomogram for predicting 5-year incidence of type 2 diabetes in a Chinese population	lack of data
Liu et al., 2018	Non-high-density lipoprotein cholesterol is more informative than traditional cholesterol indices in predicting diabetes risk for women with normal glucose tolerance	lack of data

Liu et al., 2021	The Triglyceride-Glucose Index, an Insulin Resistance Marker, Was Non-linear Associated With All-Cause and Cardiovascular Mortality in the General Population	wrong outcome
Magnusson et al., 2011	Low Plasma Level of Atrial Natriuretic Peptide Predicts Development of Diabetes: The Prospective Malmo ̈Diet and Cancer Study	not relevant factor
Mashayekhi et al., 2015	Evaluating the Performance of the Framingham Diabetes Risk Scoring Model in Canadian Electronic Medical Records	lack of data
Meigs et al., 2008	Genotype Score in Addition to Common Risk Factors for Prediction of Type 2 Diabetes	wrong population
Mora et al., 2010	Lipoprotein Particle Size and Concentration by Nuclear Magnetic Resonance and Incident Type 2 Diabetes in Women	wrong study design
Nakanishi et al., 2004	Components of the metabolic syndrome as predictors of cardiovascular disease and type 2 diabetes in middle-aged Japanese men	lack of data
Natali et al., 2017	Plasma HDL-cholesterol and triglycerides, but not LDL-cholesterol, are associated with insulin secretion in non-diabetic subjects	lack of data
Noale et al., 2006	Components of the metabolic syndrome and incidence of diabetes in elderly Italians: The Italian Longitudinal Study on Aging	lack of data
Rhee et al., 2011	Lipid profiling identifies a triacylglycerol signature of insulin resistance and improves diabetes prediction in humans	lack of data
Riediger et al., 2015	Incident diabetes, hypertension and dyslipidemia in a Manitoba First Nation	lack of data
Toro-Huamanchumo et al., 2019	Triglycerides and glucose index as an insulin resistance marker in a sample of healthy adults	wrong study design
Vega et al., 2014	Triglyceride-to-High-Density-Lipoprotein-Cholesterol Ratio Is an Index of Heart Disease Mortality and of Incidence of Type 2 Diabetes Mellitus in Men	wrong outcome
Wilson et al., 2007	Prediction of Incident Diabetes Mellitus in Middle-aged Adults	lack of data
Wong et al., 2003	The Singapore Impaired Glucose Tolerance Follow-Up Study	not relevant factor
Würtz et al., 2012	Circulating Metabolite Predictors of Glycemia in Middle-Aged Men and Women	wrong outcome
Yoon et al., 2021	The Metabolic Score for Insulin Resistance (METS-IR) as a Predictor of Incident Ischemic Heart Disease: A Longitudinal Study among Korean without Diabetes	wrong outcome

Examples for brief reasons:
wrong publication type: abstracts (any kind), reviews, guidelines, meta-analysis, protocols etc.; wrong population: pediatric population, pregnant women, adults with diabetes mellitus at baseline etc.; wrong outcome: outcome is not connected to dysglicemia (T2DM, prediabetes, impaired glucose tolerance, impaired fasting glucose, insulin resistance) etc.; wrong study design: no follow-up, TG was only measured at the diagnosis of T2DM etc.; not relevant factor: triglyceride level is not measured, triglyceride-related index is not measured, the effect of triglyceride is not investigated etc.; lack of data: lack of useful data for our analysis etc.

References used in the Supplementary Material:

1. Luo D, Wan X, Liu J, Tong T. Optimally estimating the sample mean from the sample size, median, mid-range, and/or mid-quartile range. Statistical Methods in Medical Research 2018; 27: 1785–805.
2. Shi J, Luo D, Weng H, et al. Optimally estimating the sample standard deviation from the five-number summary. Research Synthesis Methods 2020; : jrsm.1429.
3. Mantel N, Haenszel W. Statistical Aspects of the Analysis of Data From Retrospective Studies of Disease. JNCI: Journal of the National Cancer Institute 1959; 22: 719–48.
4. Robins J, Greenland S, Breslow NE. A general estimator for the variance of the mantel-haenszel odds ratio. American Journal of Epidemiology 1986; : 719–23.
5. Knapp G, Hartung J. Improved tests for a random effects meta-regression with a single covariate. Statistics in Medicine 2003; 22: 2693–710.
6. IntHout J, Ioannidis JP, Borm GF. The Hartung-Knapp-Sidik-Jonkman method for random effects meta-analysis is straightforward and considerably outperforms the standard DerSimonian-Laird method. BMC Medical Research Methodology 2014; 14: 25.
7. Jackson D, Law M, Rücker G, Schwarzer G. The Hartung-Knapp modification for random-effects meta-analysis: A useful refinement but are there any residual concerns? Statistics in Medicine 2017; 36: 3923–34.
8. Paule R, Mandel J. Consensus values and weighting factors. Journal of research of the National Bureau of Standards 1982; 87: 377–87.
9. Harrer M, Cuijpers P, A FT, Ebert DD. Doing Meta-Analysis With R: A Hands-On Guide, 1st edn. Boca Raton, FL; London: Chapman & Hall/CRC Press, 2021.
10. Veroniki AA, Jackson D, Viechtbauer W, et al. Methods to estimate the between-study variance and its uncertainty in meta-analysis. Research Synthesis Methods 2016; 7: 55–79.

11.McGuinness, LA, Higgins, JPT. Risk-of-bias VISualization (robvis): An R package and Shiny web app for visualizing risk-of-bias assessments. Res Syn Meth. 2020; 1- 7. https://doi.org/10.1002/jrsm.1411
